# Albumin and Hyaluronic Acid-Coated Superparamagnetic Iron Oxide Nanoparticles Loaded with Paclitaxel for Biomedical Applications

**DOI:** 10.3390/molecules22071030

**Published:** 2017-06-22

**Authors:** Elena Vismara, Chiara Bongio, Alessia Coletti, Ravit Edelman, Andrea Serafini, Michele Mauri, Roberto Simonutti, Sabrina Bertini, Elena Urso, Yehuda G. Assaraf, Yoav D. Livney

**Affiliations:** 1 Department of Chemistry, Materials and Chemical Engineering “G. Natta”, Politecnico di Milano, 20131 Milano, Italy; chiara.bongio@polimi.it (C.B.); alessia.coletti08@gmail.com (A.C.); andrea.serafini@polimi.it (A.S.); 2 Biotechnology and Food Engineering Department, Technion-Israel Institute of Technology, Haifa 3200000, Israel; sravitm@gmail.com (R.E.); roberto.simonutti@unimib.it (R.S.); 3 Department of Materials Science, University of Milan Bicocca, 20125 Milano, Italy; michele.mauri@mater.unimib.it; 4 Istituto scientifico di chimica e biochimica “G. Ronzoni”, 20133 Milano, Italy; bertini@ronzoni.it (S.B.); urso@ronzoni.it (E.U.); 5 Biology Department, Technion-Israel Institute of Technology, Haifa 3200000, Israel; assaraf@technion.ac.il

**Keywords:** super paramagnetic iron oxide nanoparticles (SPION), hyaluronic acid (HA), bovine serum albumin (BSA), Fe_3_O_4_·DA-BSA/HA, paclitaxel (PTX), magnetic resonance imaging (MRI)

## Abstract

Super paramagnetic iron oxide nanoparticles (SPION) were augmented by both hyaluronic acid (HA) and bovine serum albumin (BSA), each covalently conjugated to dopamine (DA) enabling their anchoring to the SPION. HA and BSA were found to simultaneously serve as stabilizing polymers of Fe_3_O_4_·DA-BSA/HA in water. Fe_3_O_4_·DA-BSA/HA efficiently entrapped and released the hydrophobic cytotoxic drug paclitaxel (PTX). The relative amount of HA and BSA modulates not only the total solubility but also the paramagnetic relaxation properties of the preparation. The entrapping of PTX did not influence the paramagnetic relaxation properties of Fe_3_O_4_·DA-BSA. Thus, by tuning the surface structure and loading, we can tune the theranostic properties of the system.

## 1. Introduction

Nanotechnology has opened the way to an incredibly high number of new composite materials. As part of the growing field of nanomedicine [1], composite nanoscale biomaterials are based on assembling nanoparticles with biomolecules. The palette of available nanoparticles is large and matched by an equally large number of biomolecules [2]. Iron oxide nanoparticles are among the most extensively studied types of nanoparticles (NPs) [3]. Superparamagnetic iron oxides nanoparticles (SPIONs), composed by magnetite (Fe_3_O_4_) or maghemite (γ-Fe_2_O_3_), have been explored in view of their potentiality in biomedical applications [4]. In fact, a key advantage of iron oxide nanoparticles in comparison to other heavy metal-based NPs is their natural integration into tissue physiology. They can be part of superparamagnetic materials, whose suspensions are generally referred to as ferrofluids, clinically investigated as MRI contrast agents [5,6]. An overview has been published both on the many possibilities to prepare SPIONs suitable for enhancing with biomolecules and for potentially engineering for biomedical applications [7]. The authors of reference [8] have already described the logic of SPION-developed biomedical applications in recent years. SPION-appropriate surface coatings can be studied for various biomedical applications, such as magnetic resonance imaging, hyperthermia, drug delivery, tissue repair, cell and tissue targeting, and transfection. Development of coated and loaded SPIONs is a promising theranostic approach. In fact, following a quite similar approach, we pursued multitasking nanostructures—using cyclodextrin as a vector of antitumor drugs and low molecular weight heparins, covalently linked to cyclodextrin—that were thought to target the nanostructures to the tumor mass, according to heparanase and growth factors inhibition mechanisms [9]. We then progressed to iron oxide and heparin-based materials to introduce the diagnostic functionality [10,11]. Since 2005, magnetic NPs have been considered of great interest for biomedical applications, and, among them, iron oxide NPs have been considered the more promising [12]. Additionally, depending on particle size, positive or negative contrast agents can be prepared [13,14]. Even though SPIONs have been clinically approved metal oxide NPs, the potential toxicity of SPION has been thoroughly investigated [14]. Although there is no intrinsic risk associated with SPION per se, adverse biological effects and safety issues could be associated with specific SPIONs. Ref. [15] argues about issues that need be addressed by the scientific community prior to approving their clinical use. We already cited that SPION-appropriate surface coatings divert SPION towards different biomedical applications. In advance, SPION coating has been used to modulate their biocompatibility and to reduce their toxicity. This is the case of Bovine Serum Albumin (BSA) protein [16]. The same authors from Reference [16] are working today on human serum albumin, for better biocompatibility, thus supporting the crucial role that protein corona can play [17].

The common challenge of engineered SPION for biomedical applications is that the material used for surface coating of the magnetic particles must not only be nontoxic and biocompatible but also enable selective targeted delivery of the particles to the location of the disease. Passive targeting of long circulating nanoparticles via the enhanced permeability and retention (EPR) effect is one of the mechanisms by which such coated nanoparticles can reach solid tumors. Decoration of engineered magnetic nanoparticles with targeting molecules that selectively bind to target proteins overexpressed on the target cells could significantly enhance the selectivity of their targeting to these cells [18,19]. Magnetic nanoparticles can deliver drugs and an external magnetic field can be applied to guide them to the target site. Different polymers/molecules have been used for nanoparticle coating to stabilize the suspensions of magnetic nanoparticles under in vitro and in vivo situation and selected proteins/targeting ligands have been used for derivatizing magnetic nanoparticles. It is noteworthy that magnetic drug targeting employing nanoparticles as carriers is a promising cancer theranostic treatment, avoiding the side effects of conventional chemotherapy [3]. Molecular imaging is one of the most promising applications of targeted iron oxide nanoparticles, and various applications using targeted iron oxide nanoparticles have been evaluated in vitro and in animal experiments [20]. Looking forward, the fact that targeted superparamagnetic iron oxide nanoparticles can be used for early detection of cancer has to be considered a plus [21]. On the other hand, the challenges associated with penetration of nanoparticles across cell and tissue barriers have been recently reviewed [22]. Therefore, current understanding led us to the conclusion that the combination of a diagnostic imaging aid along with a targeted therapeutic compound loaded onto the same NP systems is a promising route of drug delivery.

The challenge of our study was to design and build new theranostic SPION systems starting from a magnetic iron oxide core decorated with bovine serum albumin (BSA) and hyaluronic acid (HA), where each component was selected to play its peculiar role and where the SPION systems could include paclitaxel (PTX) as a drug. Hyaluronic acid (HA) is a natural linear anionic glycosaminoglycan (molecular weight (MW) 5–10^4^ kDa), composed of repeating disaccharide units of d-glucuronic acid and *N*-acetyl-d-glucosamine linked via alternating β-1,4 and β-1,3 glycosidic bonds. Owing to its biocompatibility and biodegradability, HA has been extensively investigated for medical applications. In particular, HA can specifically bind to various cancer cells that overexpress CD44 receptors. In this regard, different considerations concerning the utile length of HA oligomers must be reported. Selecting HA oligosaccharides long enough to bind to more than one CD44 receptor but too short to bind to the hyaluronic acid receptor for endocytosis (HARE) receptors in the liver (preferably <10 kDa) may enable the formation of an HA-based CD44-targeted carrier that avoids hepatic elimination while achieving tumor targeting [23]. Recently, this enhanced tumor-targeting ability as well as higher therapeutic efficacy compared to free anti-cancer agents has been reported [24]. Therefore, several HA-conjugates containing anticancer agents (such as paclitaxel or doxorubicin) have already been designed [25]. Over the years, ever new functions of the CD44 protein have been found, but the main one for our purposes is the recognition of hyaluronic acid as a component of the extracellular matrix in particular areas, such as embryonic connective tissues and the outline of invasive cancerous lesions. Most HA-drug conjugates have been developed for cancer chemotherapy as macromolecular prodrugs. HA has also been conjugated onto various drug-loaded nanoparticles for use as a targeting moiety. Hyaluronic acid was bound to the initially dextran-coated SPIONs by esterification [26].

Bovine serum albumin (BSA) protein was used to improve the colloidal stability of the nanoparticles. BSA has been one of the most extensively studied proteins because of its sequence and structural homology with human serum albumin (HSA). HSA could be considered the most suitable protein for biocompatible coating of nanoparticles for biomedical applications. Herein we used BSA as a model for HSA, with the aim of ultimately using HSA for future clinical stages. BSA is an abundant serum protein with a MW of ~66 kDa. The primary physiological function of serum albumin (SA) is to transport lipids and metabolites present in blood plasma; hence, it is a suitable carrier for hydrophobic drugs. Due to the high protein binding of various drugs, it could be used for effective incorporation of these compounds. It has an extraordinary ligand binding capacity, providing a depot for a wide variety of compounds with favorable monovalent reversible binding characteristics for transport in the body and release at the cell surface. Folate–bovine serum albumin-functionalized polymeric micelles loaded with superparamagnetic iron oxide nanoparticles have been proposed for tumor targeting and magnetic resonance imaging [27,28]. Ultrasound-triggered BSA/SPION hybrid nanoclusters investigated for liver-specific magnetic resonance imaging support the interest in the use of SA in the field of nanomedicine [29].

Paclitaxel (PTX) is a medication used to treat a number of types of cancer, including ovarian cancer, breast cancer, lung cancer, and pancreatic cancer, among others. Its clinical application is hampered by poor solubility in water and other pharmaceutically acceptable solvents (<2 μg/mL). To increase its bioavaibility, various alternative formulation approaches have been explored, including emulsions, nanoparticles, polymeric micelles, cyclodextrins, liposomes, etc. [30]. Of note, hyaluronic acid-anchored PTX nanocrystals have been found to improve chemotherapeutic efficacy and inhibit lung metastasis in tumor-bearing rat models [31].

**Scheme 1:**
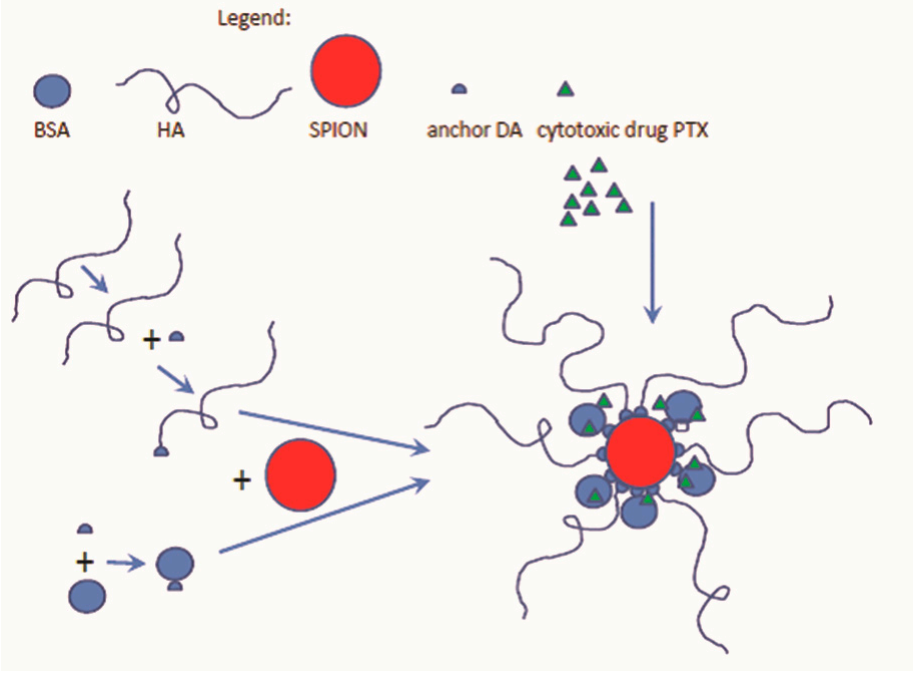
A schematic protocol of the envisioned Fe_3_O_4_·DA-BSA/HA nanoparticle, where DA means dopamine, loaded with paclitaxel (PTX), BSA—bovine serum albumin; HA—hyaluronic acid.

The aim of this study was to design and develop a new theranostic nanosystem, named Fe_3_O_4_·DA-BSA/HA, suitable for loading PTX (see Scheme 1). The iron core and HA were selected according to the specific roles they can play in therapy, diagnosis and targeting, while BSA could make stable, biocompatible and non-toxic the hybrid Fe_3_O_4_·DA-BSA/HA system. The iron core was decorated irreversibly by BSA and HA [32]. This is the reason why dopamine (DA) is a fundamental part of the system. Covalent linkage of both BSA and HA with DA was built up, DA being identified as the specific anchoring molecule to the inorganic core. BSA-DA and HA-DA were both linked to the surface of the iron core, in order to obtain a strong interaction between the bioorganic layer and the inorganic core. Scheme 1 summarizes the schematic protocol to Fe_3_O_4_·DA-BSA/HA that was thought to be as simple as possible, reproducible, and suitable for scaling up. The choice of HA and BSA was also in agreement with the final challenge of the work, that is the loading of Fe_3_O_4_·DA-BSA/HA with the real anticancer PTX drug. The rational of the PTX Fe_3_O_4_·DA-BSA/HA inclusion can also be supported by the fact that, on 6 September 2013, the Food and Drug Administration (FDA) approved PTX albumin-stabilized nanoparticle formulation and also partially by Ref. [31].

## 2. Results

### 2.1. Preparation and Characterization

#### 2.1.1. Synthesis of BSA-DA Adduct

Since DA and BSA respectively possess amine and carboxylic moieties, the common carbodimmide cross-linker chemistry was explored. The condensation between DA and BSA was performed by employing 1-ethyl-3-(3′-dimethyl-aminopropyl)-carbodiimide (EDC).

**Figure 1:**
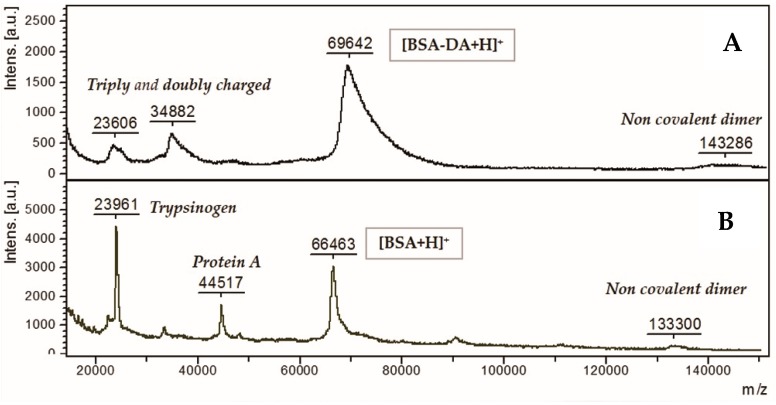
Matrix-assisted laser desorption/ionization (MALDI)-time of flight (TOF) spectra of (**A**) bovine serum albumin-dopamine (BSA-DA) and (**B**) BSA protein, protein A and trypsinogen.

The DA:BSA degree of substitution was evaluated by means of MALDI–TOF analysis (Figure 1). The spectrum shows a large peak at mass-to-charge (*m/z*) values of about 70,000 Da, corresponding to the medium average derivatization of 20:1 (20 molecules of DA for each molecule of BSA).

#### 2.1.2. Synthesis of HA-DA Adduct

Commercial low molecular weight hyaluronic acid (HA, 5400 Da) and dopamine hydrochloride were reacted to form HA-DA conjugates using EDC as the activating agent of carboxyl groups of the HA chain. The crude HA-DA was dialyzed before characterization.

Structural characterization of the starting material (HA) and the obtained HA-DA adduct was performed by LC–MS chromatography and NMR. Concerning HA, the LC–MS based method allowed the elution and identification of oligomer dispersion from 4 mer (with a molecular weight of about 700 Da) to 40 mer (with a molecular weight of about 7600 Da). Almost all the species eluting in the labelled chromatographic peaks (Figure S1) were identified; assignment is reported in Table S1. At any rate, the elution of several isomers was observed, confirming the presence of species with the same molecular weight but different sequences. As expected, the regular chains distribution on (G-ANAc)_n_, that means hyaluronic acid disaccharide repeating unit, 4-β-d-glucuronic acid (1–3) *N*-acetyl β-d-glucosamine, seems to be the most abundant, but relevant contribution arises from the other species.

**Figure 2:**
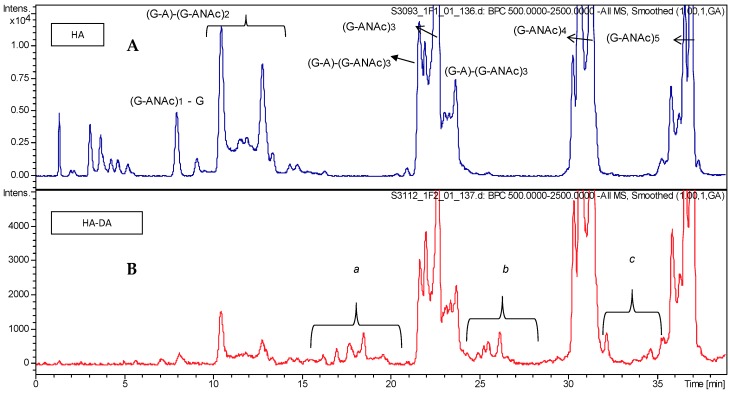
Partial HPLC–MS profiles: (**A**) intact hyaluronic acid (HA) and (**B**) HA-DA.

**Figure 3:**
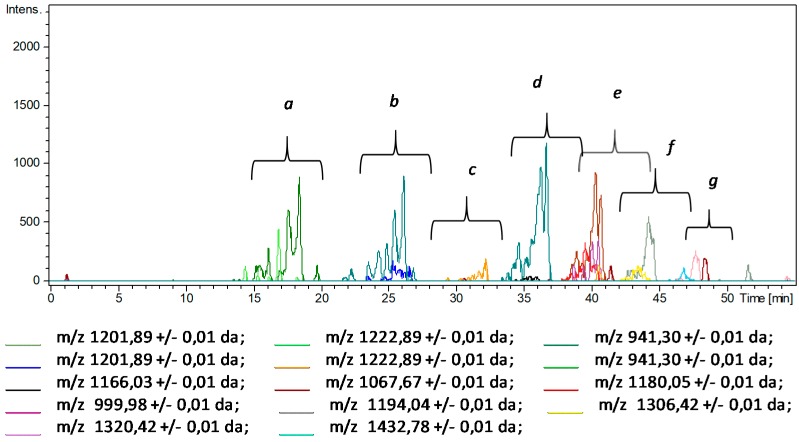
Extracted ion chromatograms (EIC) of mass signals identified as derivatized HA chains (HA-DA) ranging from 5-mer oligomers (Mw ~ 1500) to 20-mer oligomers (Mw ~ 4300) (see Table S2).

While the LC–MS analysis of intact HA provided good chromatographic separation of numerous species and their identification with very high mass accuracy, the analysis of its DA conjugate (HA-DA) showed several issues, due to the presence of unreacted starting hyaluronic acid, eventual reagents carried by the interaction with the polymer and the elution of less strongly retained derivatized oligomers under the unreacted HA chains, which cannot be quantified with this technique. Expanded portions of the chromatograms (Figure 2) allow for detecting the appearance of several mass signals corresponding to derivatized HA chains observed in the extracted ion chromatograms (Figure 3).

The elution of these derivatized components at lower retention times than the non-derivatized ones agrees with the behaviour of reversed-phase ion pair chromatography using alkylammonium as the ion pair when masking the negative charge of glucuronic acid is accomplished by reaction with DA.

Due to the extremely high complexity of the HA-DA material, we attempted to assign the structures of these signals (Table S2); data show that about the same modification, corresponding to a mass addition of about 534 Daltons, was observed in all HA components from 5 mer to 20 mer, suggesting the formation of modified HA chains containing at least three DA molecules. This first level of investigation appeared to be enough to confirm the HA derivatization with DA in the HA-DA material, as supported by parallel NMR analysis. No information can be provided via LC–MS analysis about the formation of the non-covalent HA/DA complex.

Structural characterization of HA-DA material was also performed using ^1^H- and ^13^C-NMR with mono- and bidimensional techniques such as COSY, TOCSY, DOSY, HSQC, and HMBC. Table 1 shows the chemical shifts and assignments of the signals.

**Figure 4:**
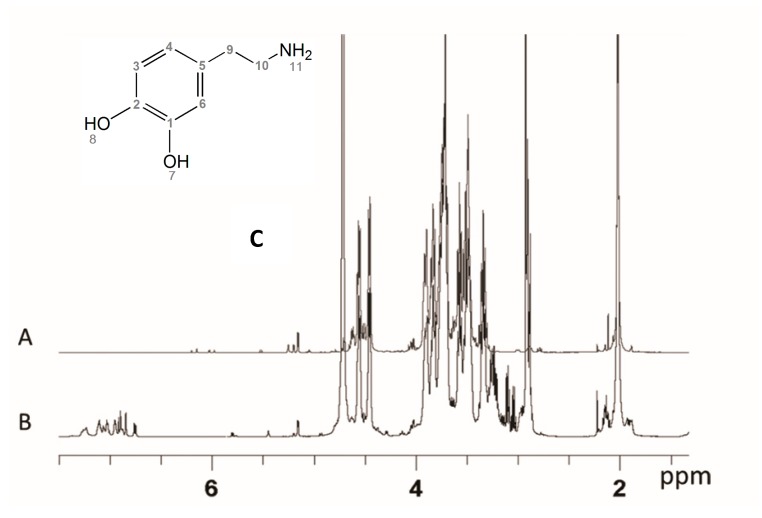
Proton spectra of HA (**A**) and HA-DA (**B**), (**C**) the DA chemical structure.

The HA ^1^H-NMR (Figure 4A) spectrum signals a low molecular weight structure. The main signals are sharper than the usual polymeric HA ones, while minor signals are due to the reducing and non-reducing terminal chain residues. Both the glucosamine and the glucuronic acid show their terminal reducing residues, suggesting a non-enzymatic chemical degradation. Integration values of easily detectable reduced end signals correspond to an average of chains centred between nine and ten disaccharide units. Free NH_2_ glucosamine is also detected, in the range of 1.5%. The spectrum of the product shows traces of minor components.

The HA-DA derivative presents a simplified ^1^H-NMR spectrum (Figure 4B) with respect to the corresponding HA spectrum induced by the derivatization procedure. The reducing anomeric pattern and other minor signals have disappeared or are significantly lowered, corresponding to a loss of the shortest HA oligomers. The quantification of reducing end anomeric signals is no longer possible due to the occurred derivatization. The main spectrum signals are unmodified, while the new signals in the expected region of the DA signals appear to be minor, in agreement with the planned derivatization degree. The ratio between the aromatic and the anomeric signals allows for estimating an average ratio of 2 DA for each 10 disaccharide building blocks. A high complexity in these spectral regions is observed.

**Figure 5:**
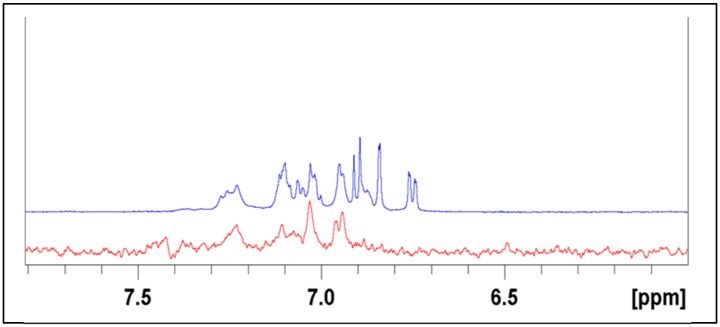
Comparison of aromatic proton sector of normal NMR spectrum (**blue**) with a DOSY section (**red**) taken in the correspondence of the polymeric chain signals.

At least three different structures are detectable at the level of the aromatic signals (Figure S2). One appears with sharp signals, and the chemical shift of the signals corresponds to the free dopamine’s surviving the dialysis step, probably because of salt effect with HA. These signals were not considered for quantitative evaluations. The two other observable structures present similar signal patterns, but both show deshielded signals, in different degrees, with respect to the free DA. Furthermore, the signals appear as broad ones, due to their incorporation in a polymeric structure. This interpretation was supported by a DOSY NMR experiment that allows identification of homogeneous molecules through their diffusion coefficients, which depend on the size and shape of the molecule. The experiment shows the polymeric chain signals containing the linked aromatic ones all aligned and having similar low diffusion coefficients, while the small components show different distributions. The discriminating spectrum of the DOSY experiment shows that the free DA component is lost while the DA-HA linked components survive (see Figure 5).

**Figure 6:**
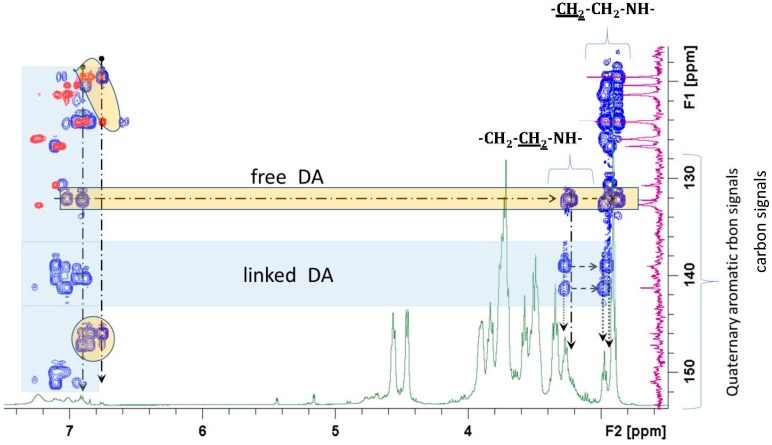
HMBC partial spectrum of HA-DA aromatic quaternary carbon correlation signals (**blue**) superimposed on the HSQC (**red**) and ^1^H spectrum (**green**). Correlations of the DA polymeric components are underlined in the light blue areas while the free form is evidenced in the yellow area.

The ^13^C spectrum and the multiple bond correlation experiment (HMBC) associated with the heteronuclear correlation (HSQC) was determinant for the identification of the quaternary carbons and their correlations with the protons of the structure. It was possible to identify the correlations, in addition to the HA structural components, of quaternary aromatic carbons of DA substituent (Figure 6) with the aliphatic CH_2_ of the linkage sequence –CH_2_–NHR. They showed chemical shifts between 3.2 and 3.3, while CH_2_ linked to the aromatic residue showed values between 2.8 and 3.0 ppm.

#### 2.1.3. Synthesis of Fe_3_O_4_·OA Nanoparticles

**Figure 7:**
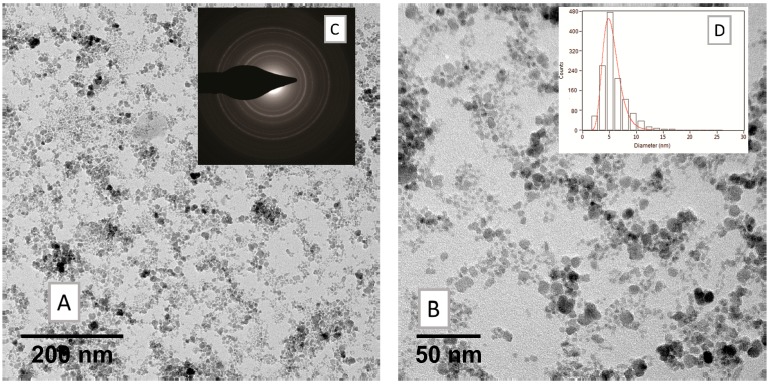
TEM measurements on nanocrystals of magnetite-oleate nanoparticles *(*Fe_3_O_4_·OA, SPION1) (**A**) low and (**B**) high magnification bright field micrographs; (**C**) relevant selected-area electron diffraction (SAED) pattern and (**D**) size distribution histogram of the nanoparticles ensemble.

The synthesis of magnetite-oleate nanoparticles *(*Fe_3_O_4_·OA, SPION1) was performed according to the following description. The magnetite was prepared by coprecipitation of a Fe^2+^ and Fe^3+^ salt solution (FeCl_2_·4H_2_O and FeCl_3_·6H_2_O, respectively) with the introduction of ammonia as an alkaline agent. No post-treatment procedure was required, since the oleic acid was introduced as a reactant during the crystallization. The FTIR spectrum (Figure S3) confirmed the formation of the product due to the presence of the bands typical for the symmetric and antisymmetric stretching vibration of oleate (1407 cm^−1^ and 1520 cm^−1^, respectively) and the signal at 600 cm^−1^, which is representative of Fe–O bond in the crystalline lattice of magnetite. Concerning the morphological characterization, dynamic light scattering (DLS) measurements showed that the averaged hydrodynamic diameter (Z_av_) of obtained oleate-nanocrystals is 31.12 ± 0.41, with a good polydispersity index (PDI) of 0.219 (Table 2). TEM images allow estimation of the size of the iron cores throughout the steps leading to the nanoassemblies. As depicted in Figure 7, their diameter is around 5 nm, and it is conserved in subsequent steps. DLS is more informative on the structure of the assemblies: the solvodynamic diameter of 30 nm of the Fe_3_O_4_·OA in hexane indicates that the loose assemblies appearing in the dry samples (Figure 7 and Table S3) are representative of the aggregation state seen in dispersion. Furthermore, different samples obtained with the same synthetic procedure over time highlighted good reproducibility of the size values.

#### 2.1.4. Synthesis of Fe_3_O_4_·DA-BSA/HA Nanoparticles

**Figure 8:**
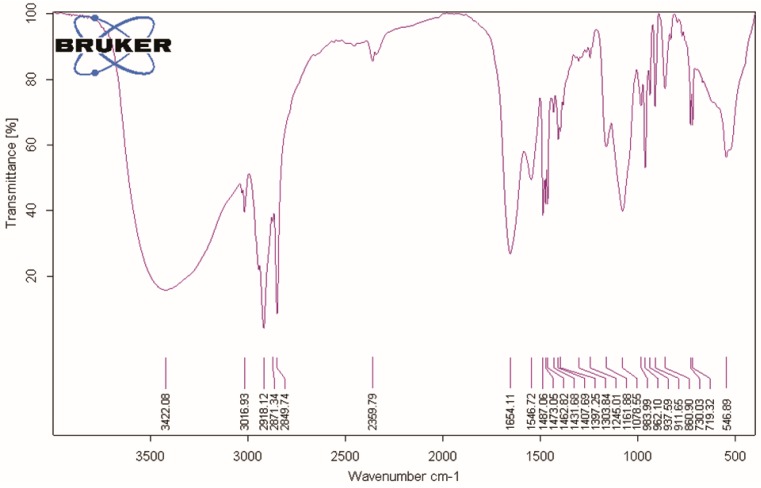
Fourier transform infrared spectroscopy (FTIR) spectrum of SPION2.

Once the BSA-DA and HA-DA adducts were prepared, they were employed in a one-pot reaction procedure by using cetyl trimethylammonium bromide (CTAB), which acts as a phase transfer agent, allowing the dispersion of magnetite-oleate nanoparticles *(*Fe_3_O_4_·OA, SPION1) in water and the contemporary ligand exchange reaction with BSA-DA and HA-DA adducts, giving sample SPION2. Figure 8 shows the FTIR spectrum of SPION2, which is representative of all Fe_3_O_4_·DA-BSA/HA.

It is possible to identify the presence of DA, HA, BSA, and cetyl trimethylammonium cation (CTA) structures and the core Fe–O by diagnostic wavenumbers per every component: ν_max_ cm^−1^: 3422.08 (O–H sym stretching, N–H stretching amide); 3016.93 (aromatic sym C–H stretching); 2918.1, 2871, 2849 (CH_3_ asymmetric stretching and –N^+^(CH_3_)_3_ symmetric stretching vibrations, CTA), 1654.11 CONH (C=O stretching); 1546.72 (aromatic sym C–C stretching); 1487.06 (benzene ring vibrations); 1473.05; 1462.82 (CH_2_ bending); 1431.68; 1397.25 (aromatic C=C bonds); 1303.84 (aromatic C–O asym bending); 1245.01 (aromatic C–O sym bending); 1161.88 (C–O alcohol); 1078.55 (C–O alcohol) 962.10 (aromatic C–C–H sym bending); 937 (–CH=CH–ring); 730.03 (C–C–C asym bending); 719.32 (C–C–C asym bending); 546.89 (Fe–O).

**Figure 9:**
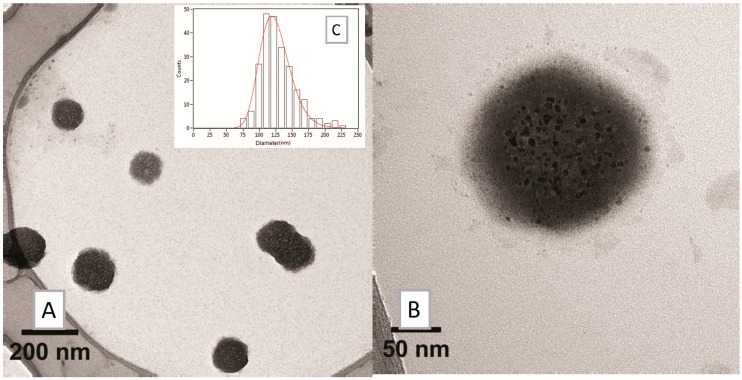
TEM analysis on SPION2: (**A**) low- and (**B**) high-magnification bright field micrographs and (**C**) size distribution histogram of the nanosystems.

The bands at 1481, 1333, and 1258 cm^−1^ have been assigned as diagnostic signals in the infrared spectrum of the bidentate ligand catechol bound to TiO_2_ [33]. Accordingly, we tentatively assigned the bands at 1487.06, 1397.25, and 1245.01 to the bidentate ligand DA bound to Fe. Bands at 2918.1, 2871, 2849, and 1462.82 were assigned to CTA, in keeping with a very recent article, “Electrochemistry and surface-enhanced Raman spectroscopy of CTAB modulated interactions of magnetic nanoparticles with biomolecules”, strictly related to our work [34]. Both TEM (Figure 9) and DLS (Z_av_ = 77.51 ± 1.32, PDI = 0.28, Table 2) depicted a significant change in the morphology of the system. The overall dimensions grew and took the shape of multiple small cores coated by shells of organic material.

**Figure 10:**
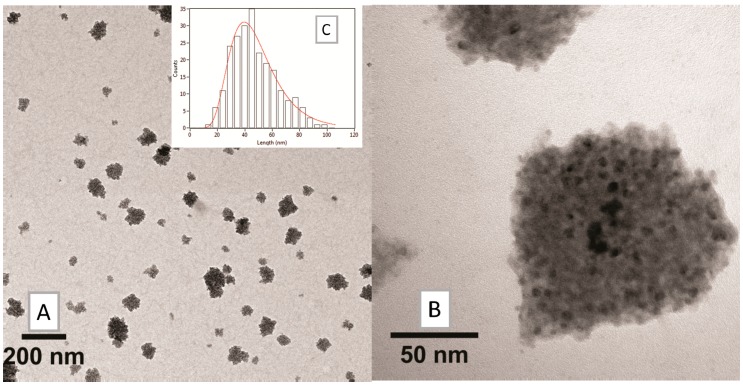
TEM analysis on SPION3: (**A**) low and (**B**) high-magnification bright field micrographs, and (**C**) size distribution histogram of the nanosystems.

**Figure 11:**
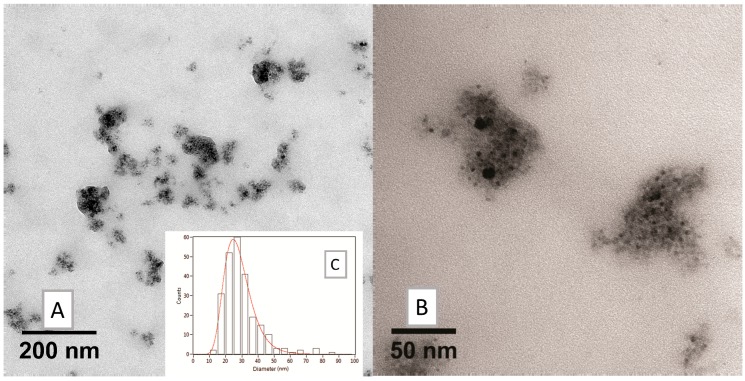
TEM analysis on SPION4: (**A**) low and (**B**) high magnification bright field micrographs, and (**C**) size distribution histogram of the nanosystems.

Since organic layer size could interfere with MRI detection by shielding the iron core, the amount of HA was decreased, giving SPION3 and SPION4 with different relative amounts of HA and BSA (20% and 10% HA, respectively, in comparison with SPION2): see the TEM images in Figure 10 and Figure 11. The choice to modulate HA content depends on the fact that HA is the real targeting component of Fe_3_O_4_·DA-BSA/HA. Hence, the availability of SPIONs 2–4 could endow Fe_3_O_4_·DA-BSA/HA with different targeting properties. Table 3 summarizes the different amounts of BSA and HA.

**Figure 12:**
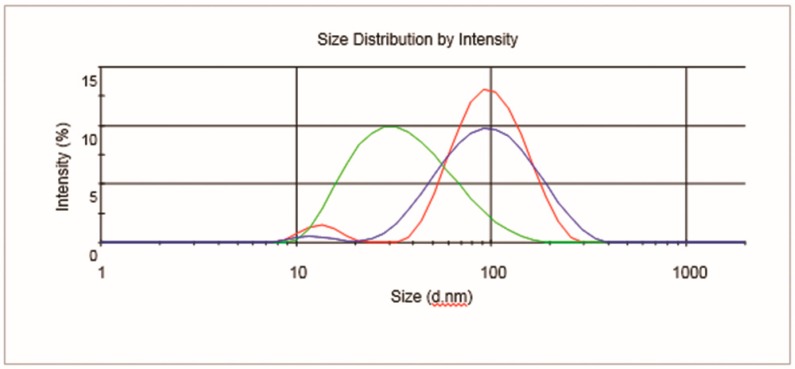
Dynamic light scattering **(**DLS) profiles of SPION1 (**green**), SPION2 (**red**), and SPION3 (**blue**).

The huge decrease of HA organic components appeared to provoke a loss in the homogeneous morphology of SPION4 (as is clearly detectable from the TEM image, Figure 11). For these reasons, SPION4 was discarded for subsequent evaluations. Concerning SPION2 and SPION3, minor differences in nanoparticle size were noticed in DLS measurements (Table 2 and Figure 12).

Concerning the evaluation of the surface charges of the nanosystem, Table 4 reports Z potential (ξ) values of different samples. Both of the functional organic molecules (HA and BSA) own an overall negative surface charge, which is slightly decreased as a result of the covalent linkage with dopamine moiety. The positive ξ values of SPIONs depend on the surface charge due to the CTA [34].

### 2.2. Inclusion of Paclitaxel and Drug Release

#### 2.2.1. Fe_3_O_4_·DA-BSA/HA Nanoparticles with Paclitaxel (PTX)

The antimitotic drug Paclitaxel (PTX) was included in both Fe_3_O_4_·DA-BSA/HA nanoparticles, resulting in samples SPION5 and SPION6, obtained from SPION2 and SPION3, respectively.

A dispersion of PTX was slowly added to progressive volumes of Fe_3_O_4_·DA-BSA/HA nanoparticles in PBS (until 1:3 *v/v*). The efficiency of the entrapment was firstly verified due to the visual disappearance of water-insoluble PTX crystals. No significant changes in the structure of the assemblies (Z_av_ in Table 2 and Figure S7), in their morphology (TEM, Figure S8) and in their superficial electric potential (ξ, Table 4) were observed.

#### 2.2.2. PTX Release Kinetics

**Figure 13:**
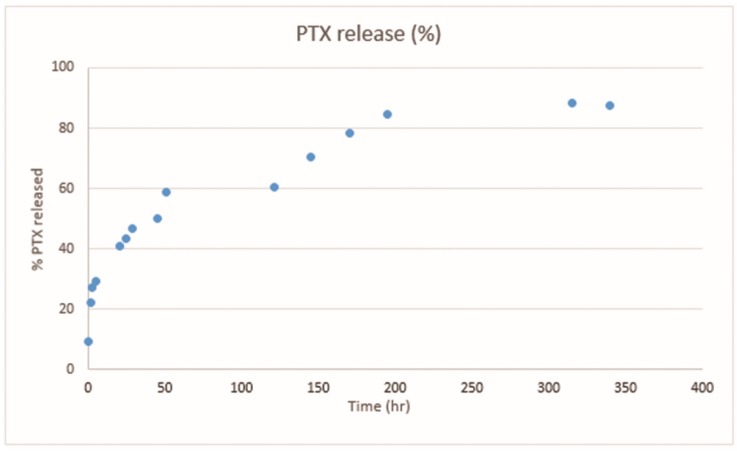
Percentage of released PTX plotted against time (hours). Data were obtained with UV-Vis experiments performed on SPION6 (see Figure S4).

SPION6 was used as the pilot nanosystem in order to test a preliminary kinetic profile of the PTX release over time. In particular, an aliquot of SPION6 colloidal solution containing PTX was submitted to a dialysis-based assay. The amount of released PTX into the dialysis solution was monitored by means of quantitative UV-Vis spectrophotometry. A gradual almost total release of the drug was observed, as detectable by plotting the percentage of released PTX versus times (Figure 13). More details are reported in the experimental part (see Section 4.4).

### 2.3. Time-domain (TD)-NMR Experiments

The solution behavior of contrast agents is often used to screen their potential before animal testing [35]. Thus, relaxation times T_1_ and T_2_ were calculated for all preparations at several different dilutions, starting from saturated dispersions of samples SPION2 and SPION3. Additionally, we tested corresponding preparations where PTX was added. For the base dispersions, we measured the amount of nanoparticles, expressed in weight, by drying. In addition, we determined the Fe atomic content of SPION2 and SPION3 by inductively coupled plasma (ICP) spectrometry. The results, expressed in % wt, are 1.38% and 5.45% for SPION2 and SPION3, respectively. Addition of the PTX was performed by adding 1 mL of PTX solution to 3 mL nanoparticle solution.

All NMR experimental data were well fitted by monomodal decay functions (Figure S5). An absence of multimodal decay indicates the system is in rapid exchange regime, with relaxation averaged by fast molecular motion between bulk solvent and nanoparticle surface. Iron nanoparticles are covered by a HA/BSA layer that makes them compatible with water as well as with each other. The coexistence of these interactions makes the grafted particles stick together into hierarchical superstructures, as is clearly detectable from TEM images (Figure 10 and Figure 11). Microscopically, they are loose assemblies where water molecules can move easily throughout the soft and hydrophilic particle organic cover, coming in proximity of the paramagnetic iron oxide cores dispersed within with high rate. This is very promising for future study on renal clearance, a desirable goal for nanoparticle design since it avoids iron buildup within the body. This kind of clearance is usually limited to nanoparticles with less than a 5.5 nm hydrodynamic radius [36], a much smaller value than that measured by DLS for the present samples. Still, it is well known that soft and flexible particles can squeeze through the renal nodules [37]. Furthermore, while the present samples are stable in PBS solution, their hierarchical nature provides the opportunity to further tune the surface for stimuli-responsive decomposition [13], ultimately separating component particles which are in the range of 3–5 nm.

**Figure 14:**
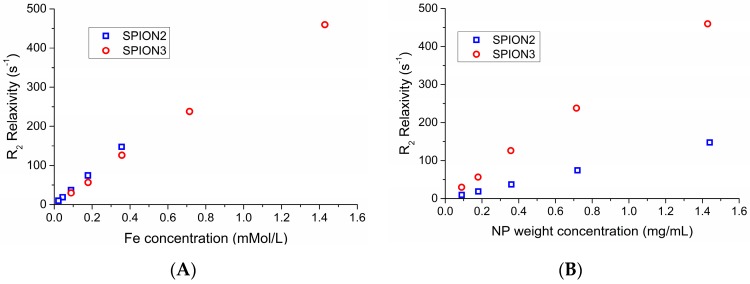
Dependence of R_2_ relaxation rates for unloaded NP over (**A**) Fe concentration and (**B**) amount of nanoparticles.

Relaxation rates R_1_ and R_2_, defined as the inverse of relaxation times T_1_ and T_2_, respectively, were plotted against the molar concentration of Fe, as exemplified by Figure 14A, which shows a comparison between the R_2_ values for samples SPION2 and SPION3. The resulting plots were highly linear (*R*^2^ > 0.99); thus, molar relaxivities r_1_ and r_2_ could be calculated as slopes of the linear fitting and are indicated in Table 5. Firstly, the high linearity indicates that the aggregates are stable, since any phenomenon of aggregation or splitting of the nanoassemblies would qualitatively change the interaction with water. The values in Fe molar concentration for the two samples span different ranges because the nanoparticle concentration in the dispersions is the same, but the nanoassemblies prepared with lower amounts of HA have a higher content in Fe cores. This is apparent in Figure 14B, where we instead plot the same relaxivity values against the amount of nanoparticles (not of Fe). Due to the higher Fe loading potential of SPION3, this compound could be the most suitable for medical applications.

On the other hand, the similarity of the values plotted against Fe concentration indicates that the particles have fundamentally the same interactions with water regardless of the BSA/HA ratio.

Later, we also verified the effect of the presence of PTX. From the data presented in Table 5 (see also figures), it is apparent that there is no effect. Similar plots were prepared for longitudinal relaxation R_1_; those values are also reported in Table 5.

The high molar relaxivity values show high promise for application as an MRI agent, since both the r_1_ and r_2_ values are comparable to top end commercial contrast agents [38]. In addition, the inclusion of PTX did not significantly influence the relaxometric parameters, suggesting theranostic applications. Since current MRI techniques are based on achieving contrast in different T_1_ and T_2_ experiments, the R_2_/R_1_ ratio is often used to evaluate the applicability of a system for use as a black or white contrast agent [39]. The present samples have values of around 5, indicating a preferential usage as a dark contrast agent, but also some possible applications for white contrast, a field currently dominated by Gd contrast agents, which have high effectivity but some well-known side effects including deposition in brain tissue and a risk of nephrogenic fibrosis.

**Figure 15:**
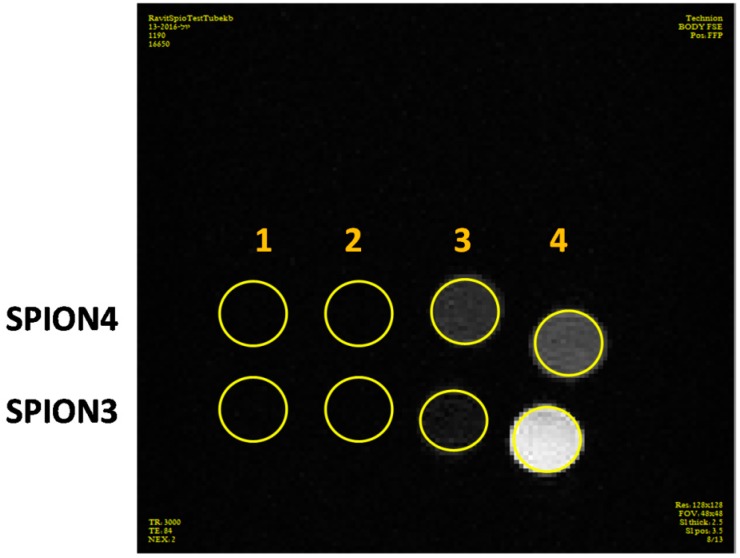
In vitro MRI T2w scans of samples SPION3 and SPION4. (**1**) Original sample; (**2**) 2-fold dilution; (**3**) 10-fold dilution; and (**4**) 50-fold dilution.

Figure 15 shows that SPIONs have a darkening effect on T2w scans, as expected, dilution of the sample with PBS decreased the signal of the SPIONs as the iron concentration decreased. The concentration of Fe_3_O_4_ in the samples was insufficient for significant signal because HA unexpectedly caused a lightening effect in the MRI, which counteracted the SPION signal reducing the signal darkening effect. A significant signal darkening was observed for the original samples of SPION3 and SPION4, but, after 50-fold dilution, the signal of SPION3 was dramatically less dark compared to SPION4, which corresponds to the fact that HA concentration of SPION4 was half the concentration of SPION3. We can conclude from this experiment that decreasing HA concentration significantly improved the ability of the SPIONs to serve as good contrast agents in MRI.

## 3. Discussion

The challenge of this work was to prepare Fe_3_O_4_·DA-BSA/HA nanoparticles (SPION samples) with the capability of loading PTX. We pursued this investigation to provide biologists and possibly clinics with new theranostic tools. We depicted SPION features and potentialities, starting with the design, passing from the synthesis to the structural and physicochemical characterization of both the final adducts and the intermediates, going on with TD-NMR investigation, and ending with the loading and release of PTX.

As anyone can recognize in current research, the nanomedicine field is becoming immeasurable. It could be compared to a dark forest where it is very difficult to find one’s bearings. As an example, a scholar intending to update the enhanced permeability and retention (EPR) of nanoparticles in tumors could not avoid considering the paper of Nichols and Bae, published in 2014, where they presented a solid overview of EPR limits and perspectives [40]. We offer these remarks to clarify the thinking we followed to project Fe_3_O_4_·DA-BSA/HA. First, we judge that the theranostic approach is still valid to fight cancer, and that its rationale is the best to pursue, as far as we know. Additionally, we decided that, in this war, our chemical approach must be not too sophisticated, while structural and physicochemical characterization needed to be as in-depth as possible. The synthetic strategy for creating SPIONs was based on well-known reactions. Dopamine (DA) has been shown to easily bind to Fe_3_O_4_ as a bidentate enediol ligand [41] and to be a robust and stable anchor group to functionalize iron oxide nanoparticles with functional molecules [42]. In Fe_3_O_4_·DA-BSA/HA, dopamine (DA) is the spacer between BSA and HA and the iron core. Furthermore, DA is very convenient as a bifunctional molecule suitable to functionalize the terminal amino group, for example by condensation with the carboxylic group of glycosaminoglycans [43] and of proteins [44]. BSA and HA were separately cross-linked to DA by an amide bond, affording BSA-DA and HA-DA, as detailed in the result and experimental sections. Of note, a dialysis procedure was used to remove nonpolymeric materials and undesired byproducts from the BSA-DA and HA-DA crude reactions. The reagents ratio employed for HA/BSA and DA crosslinking was decided with the aim of not functionalizing all the carboxylic groups of DA and HA, because in our minds HA and BSA have to maintain their biological and physical properties to act by targeting glycosaminoglycan (HA) and biocompatible protein (SA). It is well known that charges are crucial for the electrostatic interaction so important in biological environments.

A significant effort was made to characterize both the dialyzed crude reactions, putting in evidence the amido linkage formation and estimating the substitution degree of the HA and BSA carboxylic groups with DA: see Section 2.1.1 and Section 2.1.2. The medium average derivatization of BSA was estimated 20 molecules of DA for each molecule of BSA. The medium average derivatization of HA was estimated at two molecules of DA for each 10 disaccharide building blocks.

BSA-DA and HA-DA solutions were used in a one-pot reaction to perform ligand exchange on iron oleate (OA) nanoparticles, Fe_3_O_4_·OA, prepared in organic solvent [45,46]: see Section 2.1.3 and Section 2.1.4. Oleic acid coating was used to stabilize and enhance the exchange with DA adducts. Fe_3_O_4_·OA ligand exchange with DA-BSA and DA-HA provided Fe_3_O_4_·DA-BSA/HA as a bioactive adduct suitable for suspensions in buffer solution [47]. As far as we know, Fe_3_O_4_·DA-BSA/HA is a new nanostructure, in terms of its covalent DA linkages and DA chelation of Fe. HA and BSA undergo strong interactions that could be associated with significative bioactivities and synergic effects in stabilizing SPION and targeting citotoxic drugs to tumor mass. Different Fe_3_O_4_·DA-BSA/HA (SPIONs) were prepared by changing the DA-HA:DA-BSA ratios: see Table 3 and Section 4.2.4. We assume that modified DA reactivity towards Fe does not depend on the cross-linked polymers. This assumption is justified by the high affinity between DA and Fe and by the fact that steric effects are, in our opinion, not very important because of the distance between the diol and the amino group. Therefore, the initial DA-HA:DA-BSA ratio affords Fe_3_O_4_·DA-BSA/HA composition. SPIONs were all characterized by IR spectroscopy that supports the Fe_3_O_4_·DA-BSA/HA structure and gives evidence of CTA as positive counter ions of the carboxylic groups not involved in the amidation. The relevant rule that CTAB plays in modulating the interactions of magnetic nanoparticles with biomolecules [34] is noteworthy.

For convenience, the SPION2 spectrum is shown and detailed: see Figure 8. TEM images were reported in Figure 9, Figure 10 and Figure 11 and partly discussed in the results. Before further discussing SPION2-6 TEM and DLS results, let us have a look to SPION1, which is the precursor of Fe_3_O_4_·DA-BSA/HA. SPION1 could be described as a nanostructured organic salt or complex. Its morphology is shown in Figure 7. It shows iron cores surrounded by the organic layer of oleic acid. After the ligand exchange, the situation dramatically changes. The Fe_3_O_4_·DA-BSA/HA morphology resembles a pomegranate, with several iron cores covered by a single organic envelopment. The size of the iron cores does not seem to vary significantly in the different SPIONs. On the other hand, SPION size distribution depends on the different ratios between the inorganic and organic parts, as reported in Table 3. SPION2 was prepared starting from same amount of HA-DA and BSA-DA. In SPION3 preparation, the amount of HA-DA was strongly reduced, and this was even less for SPION4. SPION2 presents a much higher size distribution than SPION3; SPION4 shows the same trend. From TEM, it appears that SPION2 and SPION3 have quite the same morphology (see Figure 9 and Figure 10). We can argue that SPION2 and SPION3 are representatives of the Fe_3_O_4_·DA-BSA/HA nanosystem, where both HA-DA and HA-BSA contributes to the morphology. This is not true for SPION4 (see Figure 11). In other words, SPION4 cannot be described as a Fe_3_O_4_·DA-BSA/HA nanosystem.

SPION DLS analysis was also provided and summarized in Table 2 and Figure 12. They confirm the difference between SPION1 and the other SPIONS. The DLS data correspond well to most of the engineered SPIONs proposed in the literature. After ligand exchange, the hydrodynamic radius of the newly formed HA/BSA SPIONs increases to 70 nm. Table 2 shows that all the different Fe_3_O_4_·DA-BSA/HA fall in the range of standard SPION (50–150 nm). Noteworthy SPION that are 10–100 nm in size are considered to be optimal for intravenous administration [15]. This observation makes Fe_3_O_4_·DA-BSA/HA promising for future biomedical applications. Since the iron cores are not modified, this confirms that the assembly of large particles, as seen in Figure 9, is modulated by the interaction between biomolecular coronas and water, resulting in complex hierarchical (pomegranate) structures. Looking to the SPIONs’ morphology and size distribution, we argue that some Fe_3_O_4_·DA-BSA/HA properties could be modulated by changing the ratios between HA and DA and between them and Fe. Note especially the zeta potential (ξ) values reported in Table 4 for HA, BSA, BSA-DA, HA-DA, and SPION2, 3 and 6. The ξ values change from negative, for HA-DA and BSA-DA, to positive for SPION2 and SPION3, perfectly agreeing with surface charges due to the CTA cation [34]. The presence of CTA as counter ion can be correlated with a very good colloidal stability as observed in Ref. [34]. Furthermore, the magnetic properties of our SPIONs discussed on the base of their TD-NMR results support the biomedical diagnostic potentiality of the system Fe_3_O_4_·DA-BSA/HA plus CTA.

SPION2 and SPION3 were loaded with PTX to obtain SPION5 and SPION6, as explained in Section 2.2.1. PTX was easily dispersed by the SPION2 and SPION3 suspensions. Both of the SPION5 and SPION6 DLS and TEM measurements of the obtained drug vehicle did not highlight any significant morphological variation (see Table 2 and Figures S7 and S8). Zeta potential values were also not affected (ξ = 17.5 ± 2.21 mV, Table 4). The maintenance of size, morphology and ξ support the PTX loading within SPION2 and SPION3 affording SPION5 and SPION6. In advance, the Fe_3_O_4_·DA-BSA/HA capability to load PTX can be correlated with the PTX hydrophobic interaction with BSA. Since the nanoparticle albumin-bound paclitaxel has been discovered, many approaches have been attempted to put it in more complex systems to avoid the undesired effects of PTX anticancer drug. As a matter of fact, unfortunately the nanoparticle albumin-bound paclitaxel alone cannot solve the PTX limits and drawback. Layer-by-layer assembly of hierarchical nanoarchitectures has been investigated to enhance the systemic performance of nanoparticle albumin-bound paclitaxel [48]. The biodegradability has been pursued in one-step fabrication of agent-loaded biodegradable microspheroids for drug delivery and imaging applications [49]. A quite similar approach has been the thermoreversible gelation of poly(ethylene glycol)/poly(ester anhydride) triblock copolymer nanoparticles for injectable drug delivery systems [50]. Nanoparticle albumin-bound paclitaxel has been loaded into a nanoporous solid multistage nanovector to enhance therapeutic efficacy [51]. Finally, drug-induced self-assembly of modified albumins has been proposed for tumor-targeted combination therapy [52]. The rational of our approach to load PTX within Fe_3_O_4_·DA-BSA/HA perfectly agrees with References [48,49,50,51,52] and enhances the BSA role in Fe_3_O_4_·DA-BSA/HA structures.

As shown in Figure 13, PTX was released by SPION6 at 90% in nine days: see Section 2.2.2. In about one day, 60% of PTX was released. Both the PTX loading and the preliminary release data associated with the physicochemical characterization of SPION5 and SPION6 are strongly encouraging for the development of Fe_3_O_4_·DA-BSA/HA as PTX delivery nanosystems, where loaded PTX was made bioavailable in buffer solution and matched with the Fe_3_O_4_·DA-BSA/HA targeting potentiality according to the hope of reducing the PTX administered dose.

Section 2.3 reports the results obtained by TD-NMR experiments and highlights useful comments. In particular, the SPIONs’ magnetic properties appear crucial to recognition that Fe_3_O_4_·DA-BSA/HA can also be tested as an innovative contrast agent: see Table 5 and Figure 14. Figure 14b shows the very important result about the R_2_ relaxation of SPION2 compared with SPION3: the same NP weight concentration, but different Fe contents (SPION3 > SPION2), corresponds to a higher R_2_ relaxation for SPION3 than for SPION2.

SPIONs were reported to be excellent MRI T2 contrast agents. As excepted, in vitro T2w MRI scanning of SPION3 and SPION4 demonstrated significant dark signal having a great potential for targeted imaging. The approximate blood volume of a mouse is 77–80 µL/g weight. After IV injection (200 µL) to mice, the sample is diluted in the blood stream (1.9–2 mL) and concentrated in the tumor so the dilution of the sample is up to 10 fold. From the MRI in vitro result (Figure 15), even after 10-fold dilution the darkening effect of SPION3 and SPION4 is significant, thus we can deduct from this experiment that a sufficient signal will be presumably observed in mice. Moreover, at higher dilution, SPION4 has a better darkening signal due to its lower concentration of HA.

We can argue not only that SPION3 could be more suitable as contrast agent, but also that the synthetic strategy approached in this paper can modulate Fe_3_O_4_·DA-BSA/HA properties. The TD-NMR experiments also confirm Fe_3_O_4_·DA-BSA/HA stability, a feature that is essential for further developments. Finally, the fact that the inclusion of PTX did not at all modify the magnetic properties of Fe_3_O_4_·DA-BSA/HA strongly supports the achievement of using Fe_3_O_4_·DA-BSA/HA as theranostic agent.

## 4. Materials and Methods

### 4.1. General

Chemicals: Ferric chloride hexahydrate (FeCl_3_·6H_2_O), ferrous chloride tetrahydrate (FeCl_2_·4H_2_O), sodium chloride (NaCl), potassium chloride (KCl), sodium hydrogenphosphate (Na_2_HPO_4_), potassium dihydrogen phosphate (KH_2_PO_4_), dopamine hydrochloride (DA∙HCl), bovine serum albumine (BSA), cetyl trimethylammonium bromide (CTAB), *N*-(3 dimethylaminopropyl)-*N*′-ethylcarbodiimide hydrochloride (EDC∙HCl), hydrochloric acid (HCl, 37%), sodium hydroxide (NaOH), and paclitaxel (PTX) were purchased from Sigma Aldrich (St. Louis, MO, USA). Oleic acid ≥85% and sodium oleate were purchased from Tokyo Chemical Industry (TCI), Tokyo, Japan. Sodium hyaluronate (5400 Da) was purchased from Lifecore Biomedical, Inc., Chaska, MN, USA.

Materials: Dialysis sacks were purchased from Sigma Aldrich (MWCO: 12,000 Da) (St. Louis, MO, USA) and from SpectrumLabs (Spectra/Por 3 Dialysis Tubing, MWCO: 3500 Da). Syringe filters (25 mm, 0.22 µm) were purchased from VWR International, Milan, Italy.

### 4.2. Synthetic Procedures

#### 4.2.1. Synthesis of BSA-DA Adduct

The reaction flask was obscured to prevent denaturation of the BSA protein. First, 250 mg of BSA were dissolved in 20 mL of PBS buffer solution (NaCl 137 mM, KCl 3 mM, Na_2_HPO_4_ 10 mM and KH_2_PO_4_ 2 mM. pH = 7.4). Then 527.5 mg of 1-ethyl-3-(3′-dimethyl-aminopropyl)-carbodiimide (EDC) were added, under magnetic stirring. After a few minutes, 500 mg of dopamine hydrochloride were added to the solution, which rapidly became darker. The reaction lasted 20 h. The final mixture was dialyzed against several changes (6 × 1 L) of PBS buffer solution (MWCO: 12,000 Da). The recovered mixture was analyzed by MALDI–TOF.

#### 4.2.2. Synthesis of HA-DA Adduct

The first 250 mg of Sodium Hyaluronate (5400 Da) were dissolved with 20 mL of PBS solution (pH = 7.4). A few drops of aqueous solution of HCl (1M) were added to adjust the pH to the value of 5–5.5. Then, 120 mg of Dopamine hydrochloride and 100 mg of EDC were added. The pH of the solution rapidly increased up to 8.0, and several drops of HCl 1M were added to the solution over time in order to keep the pH values between 5.0 and 6.0 during the reaction time (3 h). After 3 h, the pH no longer increased and the reaction was considered finished. The final mixture was dialyzed against several changes (3 × 1 L) of PBS buffer solution (MWCO: 3500 Da).

#### 4.2.3. Synthesis of Fe_3_O_4_·OA Nanoparticles (SPION1)

The magnetite gel was formulated by coprecipitation. First, 3.44 g of FeCl_2_∙4H_2_O and 9.40 g of FeCl_3_∙6H_2_O were dissolved in 160 mL of distilled water under nitrogen gas with vigorous stirring, at 80 °C for 30 min. Next a mixture composed of 24 mL of NH_4_OH solution (25% NH_3_ in H_2_O), 4 mL of oleic acid, and 4 mL of acetone was slowly added to the solution. The color of the reaction mixture immediately turned to black. Stirring lasted 30 min and then the solution was heated again, to 80 °C, for 30 min. Fe_3_O_4_·OA nanoparticle precipitation was promoted by adding 200 mL of EtOH 99% and putting a magnet under the reaction flask. The transparent liquid phase was removed and the recovered black solid material was dissolved in hexane (150 mL) and reprecipitated with ethanol (200 mL), with the simultaneous promoted magnetic sedimentation. After the removal of liquid supernatant, a last washing of the nanoparticles was performed by using acetone in an ultrasonic bath for 10 min. The SPIONs were collected and dissolved in hexane, a solvent that allows their storage for a long time avoiding any aggregation phenomena. The chemical structure of dried Fe_3_O_4_·OA nanoparticles (SPION1) was investigated by FTIR analysis; TEM and DLS characterization was also performed.

#### 4.2.4. Synthesis of Fe_3_O_4_·DA-BSA/HA Nanoparticles (SPION2, SPION3, SPION4)

Once DA-BSA and DA-HA adducts were prepared, the exchange reaction between them and the oleic acid coating of nanoparticles was performed in a one-step procedure. In addition, 100 mg Fe_3_O_4_·OA (SPION1) were dispersed in 7 mL of chloroform. These dispersed nanoparticles were added to a solution of 1.33 g of cetyl trimethylammonium bromide (CTAB) in 66.5 mL of PBS buffer. Chloroform was carefully removed under reduced pressure. Suspended Fe_3_O_4_·OA nanoparticles were added, drop by drop and under vigorous stirring, to different mixtures of dissolved DA-BSA and DA-HA adducts in PBS (50:50, 80:20 and 90:10, respectively) in order to perform the ligand exchange reaction, resulting in the samples SPION2, SPION3, and SPION4, respectively. The products were soluble in PBS and the solid CTAB and the oil had to be discarded through centrifugation (9000 rpm, 30 min, r.t.). The final mixtures were dialyzed against several changes (3 × 1 L) of PBS buffer solution (MWCO: 12,000 Da). The samples were characterized with FTIR, DLS, and TEM and then submitted to further evaluations by means of TD-NMR experiments.

#### 4.2.5. Inclusion of PTX in Fe_3_O_4_·DA-BSA/HA Nanoparticles (SPION5, SPION6)

An aliquot of milky aqueous dispersion of paclitaxel (0.5 mg/mL) was added to Fe_3_O_4_·DA-BSA/HA NPs (SPION2 or SPION3) within a starting volumetric ratio of 1:1 (*v/v*). The preparation was sonicated for 10 min. Then, a further aliquot of NPs was added, with a final 1:3 (*v/v*) ratio (PTX:NPs). After a further sonication of 10 min, the system was maintained for approximately 48 h under constant stirring.

### 4.3. Characterization Methods

#### 4.3.1. Fourier Transform Infrared Spectroscopy (FTIR)

The solid phase FTIR spectra of the powdered sample with infrared grade KBr were generated using an ALPHA spectrometer (Bruker, Bremen, Germany). Data were analyzed using OPUS software, version 7.0 (Bruker, Bremen, Germany).

#### 4.3.2. Transmission Electron Microscopy (TEM)

TEM micrographs and selected area electron diffraction (SAED) patterns were acquired using a Philips CM200 Field emission gun transmission electron microscope (Koninklijke Philips N.V., Eindhoven NETHERLANDS) operating at 200 kV. The suspensions of iron oxide nanoparticles and supraparticles were deposited onto a 200 mesh holey carbon-coated copper grid and let dry a few hours before the analysis. Image analyses of TEM micrographs were performed using ImageJ software, Research Services Branch (RSB) of the National Institute of Mental Health (NIMH) [53], which is quite useful for estimating the dimension and shape of nanoparticles and supraparticles. In particular, the mean values and widths of the statistical asymmetric distribution (σ−, σ+) of particles were evaluated by fitting the experimental values with a log-normal function (Table S4).

#### 4.3.3. Dynamic Light Scattering (DLS)

Hydrodynamic diameter (Z_av_) and zeta potential (ξ) values of nanosystems were measured using the Zetasizer Nano ZS (Malvern, Worcestershire, UK) with a fixed 173° scattering angle and a 633-nm-helium-neon-laser. Data were analyzed using Zetasizer software, version 7.11 (Malvern, Worcestershire, UK). The temperature was set at 298 K. SPION1 was collected from the bulk mixture after the first dispersion with hexane and directly submitted to DLS measurement. SPION2 and SPION3 were passed through a 0.22 µm syringe filter, sonicated for 10 min, and then submitted to DLS measurements. SPION5 and SPION6 were directly collected and analyzed after 10 min of sonication.

#### 4.3.4. MALDI–TOF Measurements

Measurements of BSA samples and their corresponding dopamine derivatives were performed using an UV-MALDI–TOF Autoflex mass spectrometer (Bruker, Bremen, Germany) equipped with an ultraviolet laser (λ = 232 nm) operating in linear and positive ion mode in the mass range from 10 to 150 kDa. Each spectrum was recorded by averaging about 300 shots after appropriate mass range calibration performed with commercial standard proteins mixture (trypsinogen, protein A, albumin-bovine). The matrix solution, used either for analytes or standard proteins solution, was freshly prepared as a saturated solution of sinapinic acid (SA) in water 0.1% TFA:acetonitrile 2:1 (*v*/*v*). The analyte solutions were prepared at a concentration of 5–7 mg/mL in water (corresponding to about 100 pmol/µL of BSA protein) and mixed with the matrix solution in a 1:1 (*v*/*v*) ratio; then, 1 µL of this matrix/analyte mixture was loaded on the stainless steel probe and left to dry at room temperature.

#### 4.3.5. HPLC/Mass Spectrometry Measurements

Analyses of hyaluronic acid and its dopamine derivative were performed on an HPLC system (Platin Blue, Knauer, Berlin, Germany) coupled to ESI-Q-TOF mass spectrometer (impact II, Bruker). Because of the polyanionic nature of hyaluronic acid, chromatographic separation of the numerous components contained in extremely complex mixture of the hyaluronic acid sample was performed by ion pairing reversed-phase liquid chromatography (IP RP LC method) using alkylamine for ion pairing.

The sample solution was prepared at a concentration of 1 mg/mL. 2 µL were injected on a 2.1 × 100 mm Kinetex reversed-phase C18 column with 2.6 µm particles (Phenomenex, Aschaffenburg, Germany) hold at 35 °C and run at a flow rate of 0.15 mL/min, by the mobile phases A (dibutylamine 10 mM, acetic acid 10 mM in water) and B (dibutylamine 10 mM, acetic acid 10 mM in methanol) according to the following gradient: isocratic step at 10% B for 5 min, followed by a first linear gradient from 10% to 31% B in 30 min and a second slower gradient from 31% to 45% B in 30 min; then, column washing at 90% B for 5 min and reconditioning in the initial conditions were performed.

The mass spectrometry detector was set to negative polarity (capillary voltage: +3500 V) in the mass range from *m*/*z* 140 to *m*/*z* 2500; nitrogen gas used as nebulizer and heater gases was set at 1.8 bar and 7.0 L/min, respectively.

Mass calibration was performed by using sodium formate solution (water–isopropanol 1:1 *v/v* solution containing HCOOH 0.2% and NaOH 5 × 10^−3^ N).

#### 4.3.6. NMR Experiments

NMR spectra were recorded at 30 °C using a Bruker Avance HD spectrometer (500 MHz) equipped with a high sensitivity 5 mm TCI cryoprobe. Samples were dissolved in ^2^H_2_O (99.996%) and in 0.5 M NaCl, pH 7.4 and placed in 5-mm NMR tubes. Proton spectra were recorded with presaturation of the residual water signal with a recycle delay of 12 s and 8–16 scans. Bidimensional double quantum filter-COSY and two-dimensional Total Correlation Spectroscopy (TOCSY) spectra were acquired using 16 scans per series of 2048_320 data points with zero filling in F1 and F2 (4096_2048), and a shifted (π/2) squared cosine function was applied prior to Fourier transformation.

Heteronuclear single quantum coherence (HSQC) spectra were obtained in phase sensitivity-enhanced pure absorption mode with decoupling in the acquisition period and 24 scans while heteronuclear multiple bond correlation spectra (HMBC) were obtained with 64 scans. The matrix size of both experiments was 1024_320 data points and was zero-filled to 4096_2048 by application of a shifted (π/2) squared cosine function prior to Fourier transformation. Diffusion-ordered spectroscopy (DOSY) spectra were acquired using 32 scans and a series of 16 spin echo spectra registered with a time domain of 16 K zero-filled to 64 K.

#### 4.3.7. ICP Experiments

For the experiments, 14.4 mg of both SPION2 and SPION3 (obtained by lyofilization) were mineralized with nitric acid (HNO_3_) and subsequently analyzed using inductively coupled plasma (ICP) spectrometry. The employed instrument was a Perkin Elmer Optica 2300 (Perkin Elmer, Milan, Italy).

#### 4.3.8. Time-Domain (TD) NMR Experiments

A 0.5 T Bruker Minispec was used for relaxometry. This instrument is a low-resolution NMR spectrometer with proton larmor frequency of 19.65 MHz, equipped with static probe and a BVT3000 temperature control unit working with nitrogen gas. The temperature was calibrated using an external thermometer with an accuracy of ±1 K. The precision was 0.1 K; in these conditions, the temperature is stable within that range during the measurement.

All of the experiments were performed at a temperature of 303 K (29.85 °C), obtained using a Eurotherm nitrogen gas thermal apparatus (Como, Italy). Samples were prepared outside the NMR tube, and then 150 µL of each solution was inserted in a 10 mm o.d. tube, positioned in the magnet with the sample in the volume of maximum homogeneity of the B_0_ and B_1_ fields. All of the samples were thermalized for 10 minutes before performing the experiments.

For the π/2 and π, the pulse lengths were set to 2.07 µs and 4.15 µs, respectively. A good signal-to-noise ratio was obtained within a few scans, but due to the analytical nature of this work, each sample was measured with 128 scans during Carr–Purcell–Meiboom–Gill (CPMG) experiments and 64 scans per point during saturation recovery experiments.

For T_2_ measurements, we made use of CPMG pulse sequence with parameters optimized for analytical use at low field [54]. This sequence consisted of a first 90° pulse followed by a train of equally spaced 180° pulses. The signal is measured in the midpoint between each pair of 180° pulses and the obtained decaying curve is fitted against $A\;\exp\left(-t/T_{2}\right)$.

For T_1_, we implemented a saturation recovery sequence [55], a sequence composed by a pulse train that is optimized for defocusing the magnetization of the sample. Longitudinal magnetization was then measured using a single π/2 pulse after a set waiting time. Signal recovery as a function of time was then interpreted using the following equation:

(1) $$M_{z}=M_{0}\left[1-exp\left(-\frac{t}{T_{1}}\right)\right].$$

### 4.4. PTX-Release Experiments

SPION6 (4 mL) was placed in a dialysis sack (MWCO: 3500 Da) and dialyzed against 30 mL of mixture containing 70% PBS buffer and 30% methanol. The experiment was conducted in a shaking water bath (SW22, Julabo) at room temperature and with a shaking frequency of 100 rpm. The amount of released PTX over time was monitored through quantitative UV-Vis (UV-spectrophotometer JASCO v-650; Cremella LC, Italy), SpectraManager software (Cremella LC, Italy). Several aliquots of dialysate mixture were collected at different times and immediately submitted to UV acquisitions, and then replaced in the mixture of the dialysis experiment. The calibration curve employed for the quantitative conversion of data over time was taken from existing literature (*y* = 0.04*x* − 0.0626; *R*^2^ = 0.9931) [56,57].

### 4.5. In Vitro MRI

SPION3, SPION 4 and dilutions of those samples were scanned using a 9.4 T preclinical MRI scanner (Bruker, Bremen, Germany). Echo Time (TE) and Repetition Time (TR): TR/TE = 3000/60.

## 5. Conclusions

Our work was successful in identifying the synthetic strategy to obtain Fe_3_O_4_·DA-BSA/HA of 70–90 nm size containing different iron cores of 5 nm, quite homogenous and capable to afford well-dispersed and stable colloidal system. The synthetic strategy is quite easy, reproducible and up-scalable. Huge efforts were successfully done to modulate Fe_3_O_4_·DA-BSA/HA both in terms of the ratio between the inorganic core and the bioorganic layer, and the ratio between bovine serum albumin (BSA) and hyaluronic acid (HA) content in the bioorganic layer. Great attention was dedicated to structural and morphological characterization aspects. Fe_3_O_4_·DA-BSA/HA was capable of entrapping paclitaxel (PTX). Its physical-chemical characterization and preliminary release tests open the way to use Fe_3_O_4_·DA-BSA/HA as PTX delivery system. Noteworthy, Fe_3_O_4_·DA-BSA/HA increased the PTX bioavailability. Fe_3_O_4_·DA-BSA/HA gave good results in TD-NMR experiments to demonstrating their suitability to be developed as contrast agents in MRI.

## Figures and Tables

**Table 1:** Proton ^1^H and carbon ^13^C structure-chemical shift assignment of hyaluronic acid (HA) and HA-dopamine (DA). Italics: free DA. The numbers used for the attribution of DA signals refer to the DA structure reported in Figure 4.

HA (ppm)		Glucosamine	HA-DA (ppm)	
^1^H	^13^C		^1^H	^13^C
5.16	93.8	**A1α**	5.16	93.9
4.71	97.6	**A1β**	4.71	97.6
5.22	95.1	**A1NH_2_α**	-	-
4.56	103.3	**A1**	4.56	103.4
3.84	57.1	**A2**	3.83	57.2
3.72	85.5	**A3**	3.71	85.5
3.52	71.3	**A4**	3.51	71.3
3.48	78.3	**A5**	3.46	78.3
3.76	63.4	**A6**	3.75	63.4
3.92		**A6′**	3.90	
2.02	25.4	**ME**	2.02	25.4
-	177.8	**N-CO**	-	177.7
		**Glucuronic Acid**		
5.26	94.9	**G1_redα_**	5.20	94.9
3.01	57.1	**G2_redα_**	nd	nd
4.61	98.9	**G1_red__β_**	4.62	99.0
2.77	59.7	**G2_red__β_**	nd	nd
4.46	106.0	**G1**	4.45	105.8
3.45	75.4	**G2**	3.33	75.3
3.58	76.5	**G3**	3.56	76.5
3.74	82.8	**G4**	3.71	85.5
3.71	79.2	**G5**	3.71	79.2
-	176.9	**G6**	-	176.9
		**Dopamine**		
2.87	32.0	**CH_2_ (9)**	2.98–2.93	34.8
			2.96–2.87	
3.22	40.0	**CH_2_-Nr (10)**	3.27	43.3
			3.23	39.5
6.75	123.0	**4**	6.88	124.7
			6.93	124.2
			6.76	124.1
-	130.1	**5**		138.9
				141.3
				131.9
6.90	117.4	**3**	7.10	130.7
			7.26	125.8
			7.23	125.7
			6.91	119.4
6.85	117.4	**6**	7.02	121.2
			7.09	126.7
			6.85	119.5
-	145.0 143.8	**1 and 2**		132.1
				140.2
				149.9
				150.7
				151.1
				147.1
				145.9

**Table 2:** Zeta average (Z_av_) and polydispersity index (PDI) of nanosystems, superparamagnetic iron oxide nanoparticle (SPION) 1–5.

Sample	Z_av_ (d. nm)	PDI
SPION1	31.1 ± 0.4	0.22
SPION2	77.5 ± 1.3	0.28
SPION3	83.9 ± 0.9	0.28
SPION5	75.0 ± 1.3	0.29
SPION6	79.4 ± 0.7	0.28

**Table 3:** Different HA amounts (mg) used for the preparation of Fe_3_O_4_·DA-BSA/HA nanoparticles.

Fe_3_O_4_·OA (SPION1)	BSA	HA	Samples
100	250	250	SPION2
		50	SPION3
		25	SPION4

**Table 4:** Zeta potential (ξ) values for HA, BSA, BSA-DA, HA-DA, and SPION2, 3, and 6.

ξ (mV)	
Hyaluronic Acid (HA)	–30.8 ± 1.74
Bovine Serum Albumin (BSA)	–26.4 ± 1.51
BSA-DA	–6.14 ± 1.30
HA-DA	–2.17 ± 0.83
SPION2	+15.3 ± 1.01
SPION3	+19.2 ± 1.16
SPION6	+17.5 ± 2.21

**Table 5:** Longitudinal relaxation R_1_ of SPION2, SPION5, SPION3 and SPION6.

Sample	Composition	r_1_ ± SD (s^−1^ Mm^−1^)	r_2_ ± SD (s^−1^ Mm^−1^)	r_2_/r_1_ ± SD
SPION2	Fe_3_O_4_·DA-BSA/HA	76.8 ± 1.4	414 ± 4	5.41 ± 0.03
SPION5	SPION2 + PTX	81.2 ± 1.4	396 ± 5	4.88 ± 0.04
SPION3	Fe_3_O_4_·DA-BSA/HA _20%_	61.1 ± 0.7	321 ± 6	5.*2*6 ± 0.04
SPION6	SPION3 + PTX	62.9 ± 1.2	333 ± 6	5.29 ± 0.005

## Supplementary Materials

Supplementary materials are available online.
