# Supplementary material for: Albumin and Hyaluronic Acid-Coated Superparamagnetic Iron Oxide Nanoparticles Loaded with Paclitaxel for Biomedical Applications
Source: Molecules. 2017 Jun 22;22(7):1030. doi: 10.3390/molecules22071030 (PMC6152103; doi:10.3390/molecules22071030)
Supplement: Supplementary file 1 [file molecules-22-01030-s001.pdf]

Supplementary materials

Albumin and Hyaluronic Acid-Coated Superparamagnetic Iron Oxide Nanoparticle Loaded with Paclitaxel for Biomedical Applications

Elena Vismara[1,*], Chiara Bongio[1], Alessia Coletti[1], Ravit Edelman[5], Andrea Serafini[1], Michele Mauri[2], Roberto Simonutti[2], Sabrina Bertini[3], Elena Urso[3], Yehuda Assaraf[4], and Yoav D. Livney[5,*]

[1] Department of Chemistry, Materials and Chemical Engineering "G. Natta", Politecnico di Milano, 20131 Milano, Italy; E-mail: elena.vismara@polimi.it

[2] Department of Materials Science, University of Milan Bicocca, 20125 Milano, Italy; E-mail: michele.mauri@mater.unimib.it

[3] Istituto scientifico di chimica e biochimica "G. Ronzoni", 20133 Milano, Italy; E-mail: bertini@ronzoni.it

[4] Biology Department, Technion-Israel Institute of Technology, Haifa, 3200000, Israel; E-mail: assaraf@technion.ac.il

[5] Biotechnology and Food Engineering Department, Technion-Israel Institute of Technology, Haifa, 3200000, Israel; E-mail: livney@technion.ac.il

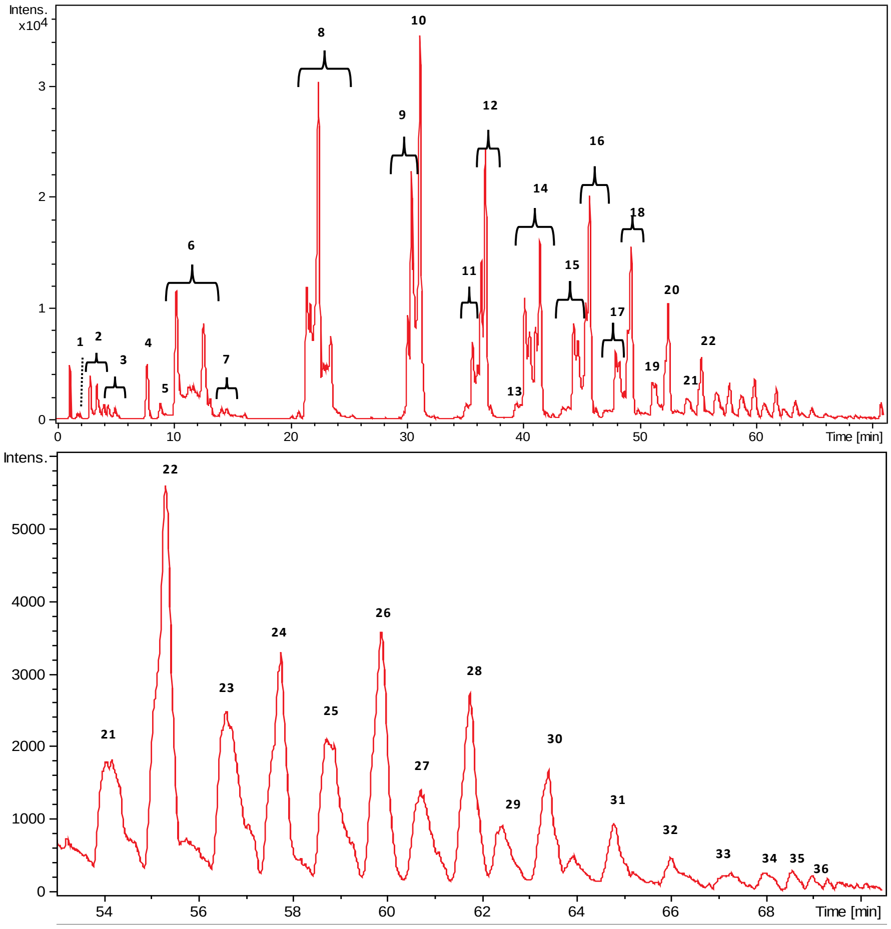

Figure S1. HPLC/ESI-Q -TOF mass spectrometry profile of intact hyaluronic acid: the whole chromatogram (upper panel) and the expanded chromatogram portion at higher retention times (lower panel). *Assignment of the labelled peaks are reported in table S1*).

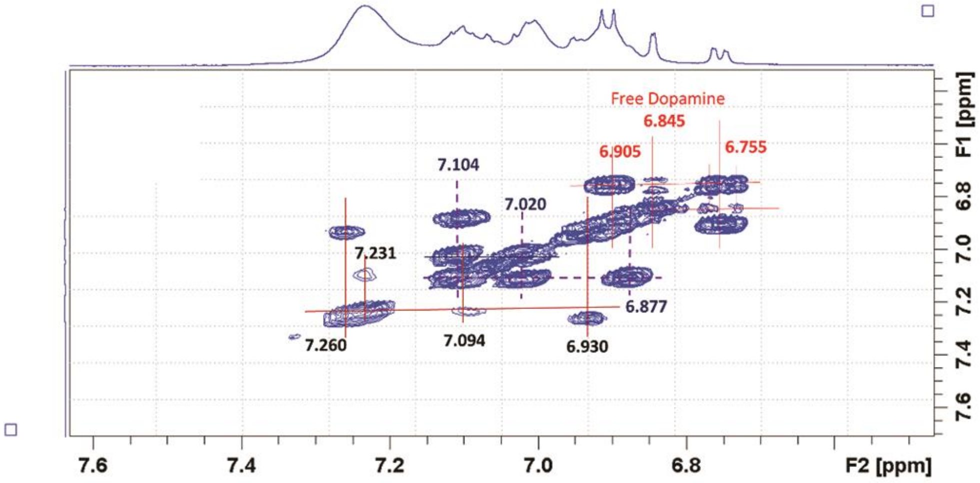

Figure S2. COSY spectrum, expansion of aromatic signals. Three correlation systems are well evident, a signal belonging to a fourth one is also shown.

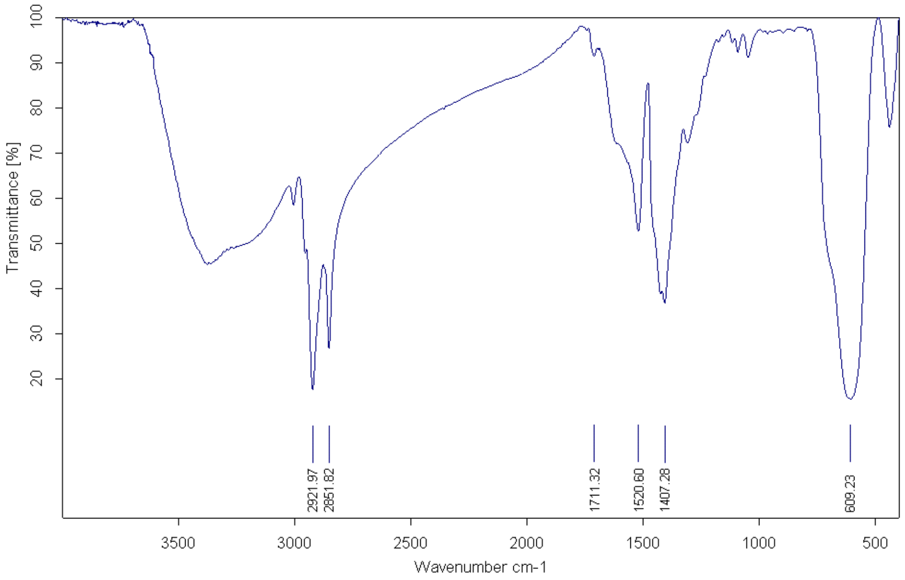

Figure S3. FT-IR spectrum of sample **SPION1**

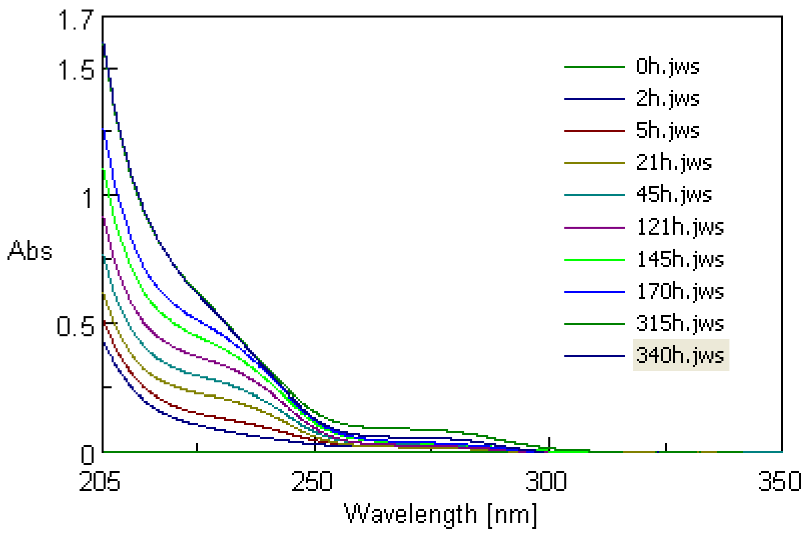

Figure S4. UV-vis spectra of dialysate buffer at selected times

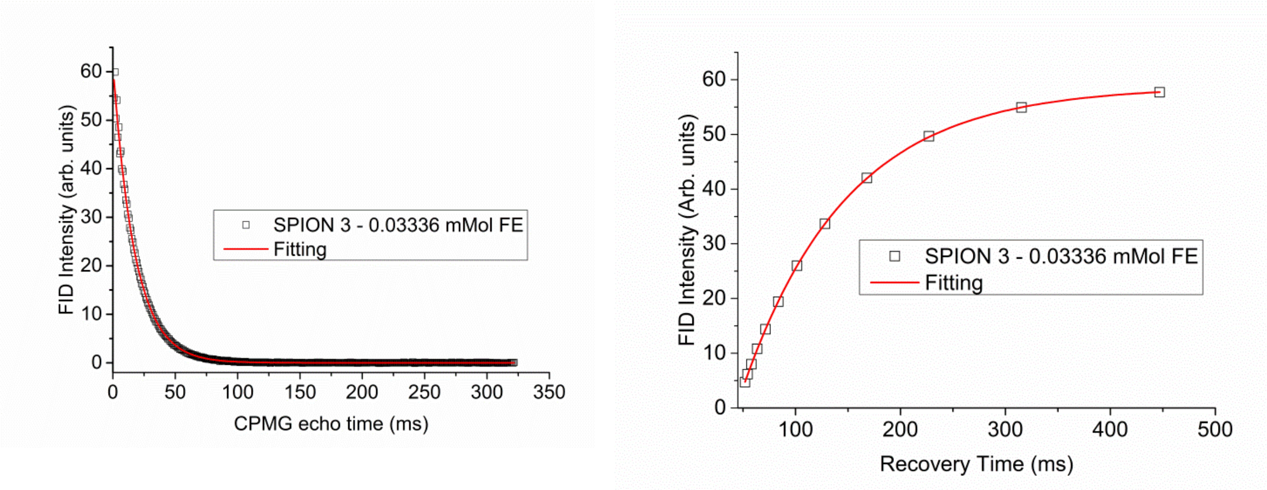

Figure S5: Example of monomodal fitting for sample **SPION3** diluted to 0.125 of the initial concentration. On the left, fitting of CPMG data with an exponential decay function. On the right, fitting of the saturation recovery curve.

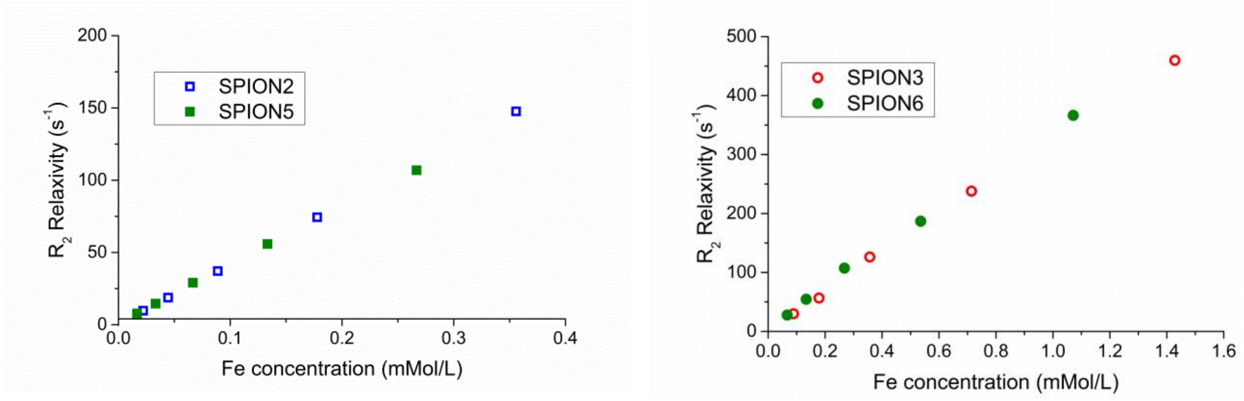

Figure S6: Comparison between analogous particles before and after addition of PTX.

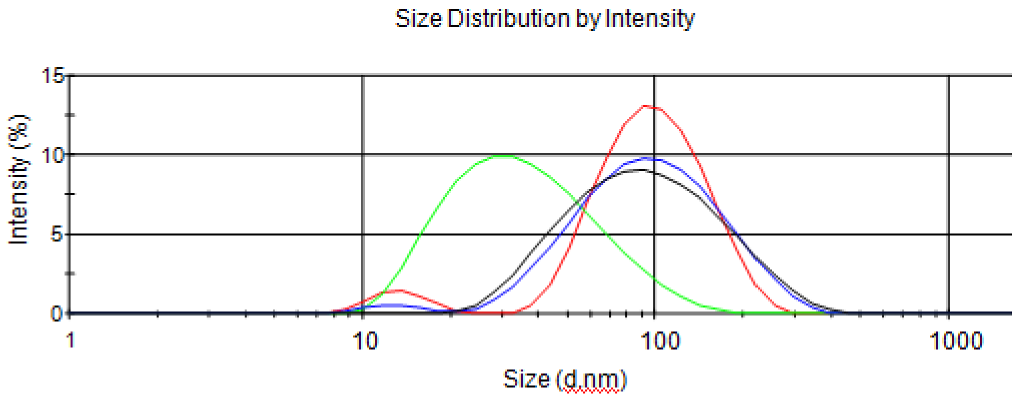

Figure S7. DLS profiles of **SPION1** (**green**), **SPION2** (**red**), **SPION3** (**blue**) and **SPION6** (**black**)

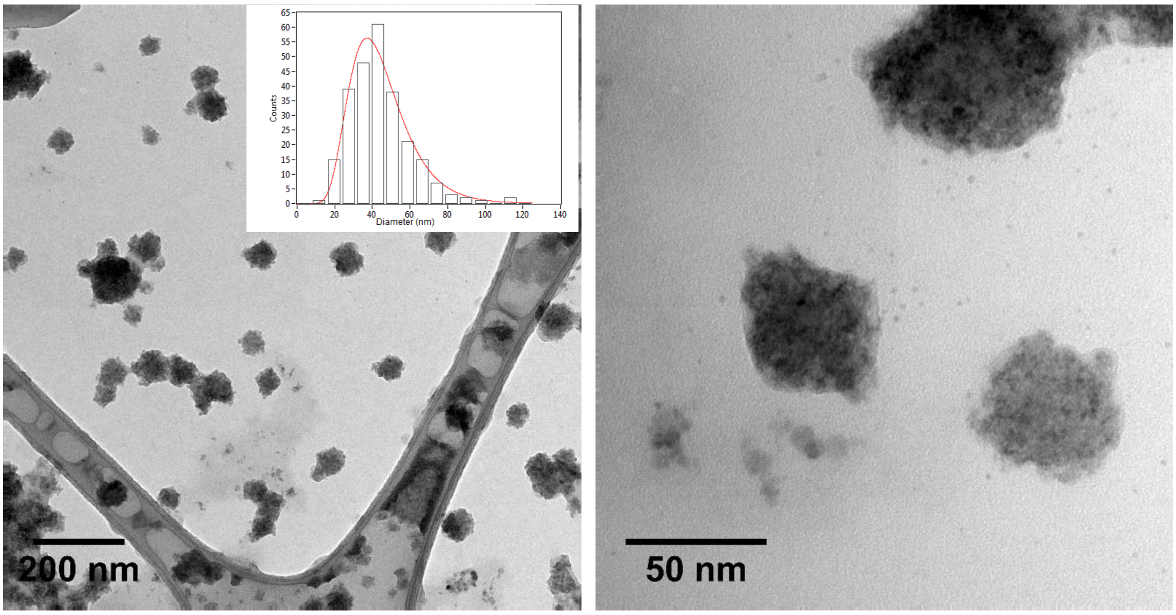

Figure S8. TEM analysis on supra-particles (**SPION6**): Low (A) and high (B) magnification bright field micrographs and size distribution histogram of the supra-particles ensemble (C).

Table S1. Experimental and theoretical masses values for identified hyaluronic oligosaccharides.

Regular chains where all the glucosamine residues are acetylated (**green** structures); components where one and two glucosamine residues are not acetylated (**red** and **black** structures, respectively); odd species exhibiting a glucuronic acid both at the reducing and at the non reducing end. Regarding to the oligomers containing non N-acetylated glucosamine residues, the extremely high mass accuracy of the MS detector allow to determine the type and number of monomers of each component, but doesn't provide the exact sequence.

Peak N.	Experimental m/z	z	M	Structure hypothesis	Theoretical m/z	Error (ppm)	
1	691.2046	-1	692.2	(G-A)$_2$	691.2040	0.9	4 mer
2	733.2142	-1	734.2	G-A- G-A$_{NAc}$	733.2145	0.4	4 mer
3	534.6551	-2	1071.3	(G-A)$_2$- G-A$_{NAc}$	534.6535	2.3	6 mer
4	572.1462	-1	573.1	(G-A$_{NAc}$)$_1$-G	572.1457	0.9	3 mer
5	775.2243	-1	776.2	(G-A$_{NAc}$)$_2$	775.2251	1.0	4 mer
6	555.6604	-2	1113.2	(G-A)-(G-A$_{NAc}$)$_2$	555.6588	2.9	6 mer
7	724.2099	-2	1450.4	(G-A)$_2$-(G-A$_{NAc}$)$_2$	724.2093	0.8	8 mer
8	576.6654	-2	1155.3	(G-A$_{NAc}$)$_3$	576.6641	2.2	6 mer
	+ 745.2154	-2	1492.4	(G-A)-(G-A$_{NAc}$)$_3$	745.2145	1.2	8 mer
9	934.7700	-2	1871.5	(G-A)-(G-A$_{NAc}$)$_4$	934.7703	0.3	10 mer
10	934.7705	-2	1871.5	(G-A)-(G-A$_{NAc}$)$_4$	934.7703	0.2	10 mer
	+ 664.6813	-2	1331.4	(G-A$_{NAc}$)$_3$-G	664.6801	1.8	7 mer
	+ 766.2206	-2	1534.4	(G-A$_{NAc}$)$_4$	766.2198	1.0	8 mer
11	861.5831	-3	2588.7	(G-A)$_2$-(G-A$_{NAc}$)$_5$	861.5815	1.9	14 mer
	+ 749.2163	-3	2250.6	(G-A)$_1$-(G-A$_{NAc}$)$_5$	749.2145	2.4	12 mer
12	854.2366	-2	1710.5	(G-A$_{NAc}$)$_4$-G	854.2359	0.8	9 mer
	+ 955.7757	-2	1913.6	(G-A$_{NAc}$)$_5$	955.7756	0.1	10 mer
13	987.9534	-3	2966.9	(G-A)$_2$-(G-A$_{NAc}$)$_6$	987.9520	1.4	16 mer
14	763.2198	-3	2292.7	(G-A$_{NAc}$)$_6$	763.2181	2.2	12 mer
	+ 875.5864	-3	2629.8	(G-A)$_1$-(G-A$_{NAc}$)$_6$	875.5850	1.6	14 mer
15	1001.9574	-3	3008.9	(G-A)$_1$-(G-A$_{NAc}$)$_7$	1001.9555	1.9	16 mer
16	889.5904	-3	2671.8	(G-A$_{NAc}$)$_7$	889.5886	2.0	14 mer
	+ 821.8970	-3	2468.7	(G-A$_{NAc}$)$_6$-G	821.8954	1.9	13 mer
17	1128.3276	-3	3388.0	(G-A)$_1$-(G-A$_{NAc}$)$_8$	1128.3260	1.4	18 mer
	1060.6347	-3	3184.9	(G-A)$_1$-(G-A$_{NAc}$)$_7$-G	1060.6333	1.3	17 mer
18	1015.9610	-3	3050.9	(G-A$_{NAc}$)$_8$	1015.9590	2.0	16 mer
	+ 948.2670	-3	2847.8	(G-A$_{NAc}$)$_7$-G	948.2659	1.2	15 mer
19	1254.6981	-3	3767.1	(G-A)$_1$-(G-A$_{NAc}$)$_9$	1254.6965	1.3	20 mer
	+ 1187.0052	-3	3564.0	(G-A)$_1$-(G-A$_{NAc}$)$_8$-G	1187.0034	1.5	19 mer
20	1142.3308	-3	3430.0	(G-A$_{NAc}$)$_9$	1142.3295	1.1	18 mer
	+ 1074.6378	-3	3226.9	(G-A$_{NAc}$)$_8$-G	1074.6364	1.3	17 mer
21	1035.5505	-4	4146.2	(G-A)$_1$-(G-A$_{NAc}$)$_{10}$	1035.5481	2.3	22 mer
	+ 1381.0697	-4	5528.4	*n.d.* [a]			
22	1268.7015	-4	5078.8	*n.d.* [a]			
	+ 1201.0080	-4	4808.0	*n.d.* [a]			
23	1130.3278	-4	4525.3	(G-A)$_1$-(G-A$_{NAc}$)$_{11}$	1130.3260	1.6	24 mer
24	1046.0526	-4	4188.2	(G-A$_{NAc}$)$_{11}$	1046.0508	1.7	22 mer
	+ 1395.0722	-4	5584.3	*n.d.* [a]			
25	1225.1068	-4	4904.4	(G-A)$_1$-(G-A$_{NAc}$)$_{12}$	1225.1039	2.4	26 mer
26	1140.8313	-4	4571.3	(G-A$_{NAc}$)$_{12}$	1140.8286	2.4	24 mer
	+ 1090.0609	-4	4364.2	(G-A$_{NAc}$)$_{11}$-G	1090.0588	1.9	
27	1319.8853	-4	5283.6	(G-A)$_1$-(G-A$_{NAc}$)$_{13}$	1319.8817	2.7	23 mer
	+ 1269.1113	-4	5080.4	*n.d.* [a]			
28	1235.6101	-4	4946.4	(G-A$_{NAc}$)$_{13}$	1235.6065	2.9	26 mer
	+ 1184.8404	-4	4743.4	(G-A$_{NAc}$)$_{12}$-G	1184.8367	3.1	25 mer
29	1414.6649	-4	5662.6	(G-A)$_1$-(G-A$_{NAc}$)$_{14}$	1414.6596	3.7	30 mer
30	1330.3883	-4	5325.6	(G-A$_{NAc}$)$_{14}$	1330.3844	2.9	28 mer
	+ 1279.6168	-4	5122.5	(G-A$_{NAc}$)$_{13}$-G	1279.6145	1.8	27 mer
31	1425.1662	-4	5704.7	(G-A$_{NAc}$)$_{15}$	1425.1622	2.8	30 mer
32	1519.9445	-4	6083.8	(G-A$_{NAc}$)$_{16}$	1519.9401	2.9	32 mer
33	1434.8190	-5	7179.0	(G-A)$_1$-(G-A$_{NAc}$)$_{18}$	1434.8152	2.6	38 mer
34	1510.6503	-5	7558.3	(G-A)$_1$-(G-A$_{NAc}$)$_{19}$	1510.6375	8.5	40 mer
	+ 1367.4001	-5	6842.0	(G-A$_{NAc}$)$_{18}$	1367.3950	3.7	36 mer
35	1443.2228	-5	7222.1	(G-A$_{NAc}$)$_{19}$	1443.2173	3.8	38 mer
36	1519.0577	-5	7600.5	(G-A$_{NAc}$)$_{20}$	1519.0396	12	40 mer

G: Glucuronic acid; A: glucosamine; A$_{NAc}$: N-acetyl glucosamine. Underlined value: main species.

Table S2. Experimental masses values for identified derivatized hyaluronic oligosaccharides.

Peaks group	Experimental m/z	Charge state (z)	Molecular weight of derivatized oligomer	Mass interpretation	Molecular weight of intact HA oligomer
a	742.2520	-2	1486.5	$(G\text{-}A_{NAc})_2$-G + X	952.4
	843.7902	-2	1689.6	$(G\text{-}A_{NAc})_3$ + X	1155.3
b	1201.8923	-2	2405.8	$(G\text{-}A_{NAc})_4$-G –A + X	1871.5
	1033.3466	-2	2068.7	$(G\text{-}A_{NAc})_4$ + X	1534.4
c	1222.8952	-2	2447.8	$(G\text{-}A_{NAc})_5$ + X	1913.6
d	941.3048	-3	2826.9	$(G\text{-}A_{NAc})_6$ + X	2292.7
	1166.0331	-3	3501.0	$(G\text{-}A_{NAc})_6$ – $(G\text{-}A)_2$ + X	2966.9
e	1067.6738	-3	3206.0	$(G\text{-}A_{NAc})_7$ + X	2671.8
	1180.0499	-3	3543.2	$(G\text{-}A_{NAc})_7$ – G-A + X	3008.9
	999.9759	-2	2001.95	n.d	-
f	1194.0448	-3	3585.1	$(G\text{-}A_{NAc})_8$ + X	3050.9
	1306.4210	-3	3922.3	$(G\text{-}A_{NAc})_8$ – G-A + X	3388.0
g	1320.4192	-3	3964.2	$(G\text{-}A_{NAc})_9$ + X	3430.0
	1432.7853	-3	4301.4	$(G\text{-}A_{NAc})_9$ – G-A + X	3767.1

where X corresponding to a mass difference of about 534.3, was identified as $C_{32}H_{30}N_4O_4$ (theoretical value of neutral monoisotopic mass: 534.2267) suggesting the formation of derivatized HA chains containing at least three dopamine molecules.

Table S3: Comparison between lattice d-spacing extracted from SAED ring pattern (**Figure 7**, TEM of **SPION1**) and the standard atomic spacings for Fe_3O_4 along with their respective hkl indexes from the PDF database.

Ring	h	k	l	d-spacing (Å)	
				SPION1	Standard Fe_3O_4
**1**	**2**	**2**	**0**	2.97	2.97
**2**	**3**	**1**	**1**	2.54	2.53
**3**	**4**	**0**	**0**	2.11	2.10
**4**	**4**	**2**	**2**	1.72	1.71
**5**	**5**	**1**	**1**	1.62	1.61
**6**	**4**	**4**	**0**	1.49	1.48

Table S4: Dimensional analysis data of iron oxide nanocrystals and final nanosystems.

Sample	Counted NPs	NPs mean diameter (nm)	σ- (nm)	σ+ (nm)	Counted nanosystems	Particles mean diameter (nm)	σ- (nm)	σ+ (nm)
**SPION1**	1280	4.7	1.6	1.6				
**SPION2**					235	119	27	35
**SPION3**					251	40	16	28
**SPION4**					241	25	9	13
**SPION6**					253	37	14	24
